# Adapting Experience‐Based Co‐Design to Disability Research: Co‐Producing the CycLink Co‐Design Study

**DOI:** 10.1111/hex.70276

**Published:** 2025-04-28

**Authors:** John Joseph Carey, Alicia Spittle, Christine Imms, Nora Shields, Margaret Wallen, Finn O'Keefe, Miriam Joy Yates, Holly Skilbeck, Rachel Toovey

**Affiliations:** ^1^ Department of Physiotherapy The University of Melbourne Melbourne Australia; ^2^ Murdoch Children's Research Institute Melbourne Australia; ^3^ Department of Paediatrics The University of Melbourne Melbourne Australia; ^4^ Olga Tennison Autism Research Centre, School of Psychology and Public Health La Trobe University Melbourne Australia; ^5^ Australian Catholic University (North Sydney Campus) Sydney Australia

**Keywords:** children and young people, co‐production, co‐researcher, cycling, disability, experience‐based co‐design

## Abstract

**Introduction:**

Participatory methods like experience‐based co‐design (EBCD) can be used to develop complex interventions, but may need adaptations when co‐designers include young people with disability, parents and community partners. We aimed to adapt EBCD through co‐production by involving people with lived experience of disability as co‐researchers. This paper reports the co‐produced protocol and reflects on co‐researchers' contributions.

**Methods:**

Guided by a six‐stage co‐production process, we formed a team of co‐researchers, academic researchers, co‐design convenors and evaluators. A five‐person steering group, comprising three co‐researchers and two academic researchers, led decision‐making and project oversight. We communicated via videoconferencing, phone and email. Briefing documents, meeting minutes and diaries supported our reflections and reporting.

**Results:**

We adapted EBCD to include people with disability through creative online methods and co‐produced a two‐part ‘CycLink Co‐design Study’ protocol. Part 1 proposed using EBCD to design principles for a community cycling intervention (CycLink). Part 2 planned a mixed‐methods evaluation of our adapted EBCD. Co‐researchers influenced participant choice and accessibility by developing phased involvement options, inclusive consent processes and adapted research materials. Interpretative support during qualitative analysis improved the relevance and reflexive rigour of findings. However, resource constraints limited co‐researcher involvement in conducting EBCD activities.

**Conclusion:**

Co‐production enabled us to adapt EBCD for people with diverse support needs and invite under‐represented populations (e.g., young people with childhood‐onset disability) to co‐design. Cumulative adjustments resulted from our disability expertise, guidelines and approaches facilitating co‐designers' opportunities to engage. Future studies should consider early and ongoing co‐researcher involvement within both processes.

**Patient or Public Contribution:**

Two adults with disability and a parent of a young child with disability joined our team as co‐researchers. Co‐researchers valued flexible involvement, which ranged from consultative to collaborative. Co‐researchers' experiential expertise influenced the relevance of project materials and qualitative findings. We reported on co‐researcher involvement through the Guidance for Reporting Involvement of Patients and the Public Version 2 Short Form (GRIPP2‐SF) [1] (Supplemental File S1—Section A, Table S1).

## 1 Introduction

Partnership‐based approaches [2] to intervention development, such as co‐design, co‐creation and co‐production, offer collaborative ways to involve consumers and community partners in developing complex interventions. Complex intervention development is a broad process that includes design as a distinct time point [3]. Design involves conceptualising intervention elements [4], including conceptual goals, components and principles. To date, people with disability have lacked opportunities to collaborate on health, fitness or active leisure intervention development or design [5].

Many children and young adults (herein referred to as young people) with disability face additional barriers to learn and participate in cycling [6, 7]. Barriers can include individual or environmental challenges and/or access to assistive technology and personnel [6]. These factors lead many young people with disability to seek interventions to support cycling goals. Evidence suggests that beginner programmes develop emergent skills [8, 9, 10, 11] but rarely link riders to community practice [12]. Strategies to develop advanced skills (e.g., navigate foot, bike or car traffic) and sustain community participation are lacking [6, 10]. Therefore, we planned to collaborate with consumers and community partners to co‐design a community‐based programme.

In our context, consumers include people with disability and immediate caregivers (e.g., parents). We identified people with disability as those with ‘long‐term physical, mental, intellectual or sensory impairments, which, in interaction with various barriers, may hinder their full and effective participation in society on an equal basis with others’ [13]. Community partners included cycling advocacy representatives and cross‐sector providers in allied health, education, sport and recreation. We acknowledged that the terms co‐production, co‐creation and co‐design are often used interchangeably [14] within partnership‐based approaches. While these approaches always intend to collaborate, recent reviews suggest variations in the origins and applications of the respective processes [14, 15]. We differentiated between co‐production and co‐design as two distinct processes and used each process with people with lived experience of disability for different purposes.

We defined co‐design as a solution‐oriented process which brings consumers, community partners and researchers together to ‘solve a particular problem or challenge’ [16] or enhance the ‘human experience’ [17, 18]. We envisaged using co‐design to design the principles of a novel cycling programme alongside young people with disability (aged 8–30 years), parents and community partners. Given the paucity of evidence exploring young people's experiences of learning to cycle or using beginner programmes, we sought an experience‐based approach. Experience‐based co‐design (EBCD) is an established process originating from healthcare quality improvement [19]. EBCD uses qualitative methods conducted over ‘two essential phases’ of experience gathering and co‐design [20]. A preceding planning phase and celebration event are also important, but often overlooked [20]. Several steps are embedded into each phase, ranging from observations, interviews, touchpoint identification (i.e., an interaction or feature of a service or product that affects overall experience), a feedback video and workshops [21]. EBCD aims to identify emotional touchpoints in a consumer's journey and generate change initiatives through consumer and provider collaboration. Traditionally, EBCD has been used to improve health services or care pathways, and researchers [20, 22] have recently identified potential applications for intervention design.

We anticipated that adaptations would be required to extend EBCD to our research and create an inclusive setting for co‐designers with diverse abilities. For us, inclusivity meant enabling people from diverse backgrounds to participate meaningfully [23]. We operationalised inclusivity by considering roles, methods and approaches that enable participation and offer a sense of belonging [24]. Collaborative roles empower consumers to become ‘makers and shakers’ [25] rather than passive recipients. Methods include democratic procedures, accessible materials and flexible involvement opportunities. Our approach to disability was influenced by inclusive research methods [26, 27], strengths‐based practice [28] and contextually important frameworks [29, 30]. Therefore, we superimposed co‐production [31] as an inclusive method [26] across our entire research process. We were guided by Strnadová's co‐production definition, as a ‘process of collaboration and collective decision making’ where ‘all consumers have a role in knowledge creation’ [31]. In co‐production, consumers join the research team as co‐researchers offering ‘an equal but different contribution’ to research planning, conducting and reporting [31]. Involvement spans a continuum from consultative feedback to collaborative contributions and consumer‐led roles [26]. Impact helps describe the value of co‐researcher contributions on research decisions and outputs [32]. Co‐production is driven by the principles of diversity, power sharing, accessibility, reciprocity, flexibility and transparency [31]. Using co‐production as our overarching process, we perceived advantages in preparing EBCD resources, offering steering group oversight and supporting qualitative methods.

The purpose of this paper is to describe our co‐produced protocol and report co‐researchers' involvement. We aim to describe:

A. how EBCD was adapted to our context;
B. the co‐produced EBCD study protocol and
C. the involvement and impact of co‐researchers throughout co‐production.

## 2 Materials and Methods

### 2.1 Context

This study forms the development phase of a larger project called ‘CycLink’. CycLink aims to connect young people with cycling goals to local cycling opportunities through a participation‐based programme. Early conceptualisation of CycLink was initiated by academic researchers (R.T. and J.J.C.) and drew upon a literature review, cross‐sector engagement and a randomised controlled trial for young people with cerebral palsy [33]. While the trial intervention was effective at achieving two‐wheel cycling goals [9], gaps were evident for adapted bike users and consumer involvement. Therefore, we engaged with five consumers (*n* = 2 adults with disability and *n* = 3 parents) to gauge the suitability of EBCD and apply for a grant. This early engagement informed us that involving consumers should extend to ‘all parts of the research’, including both the research process (i.e., co‐production) and CycLink's design (i.e., co‐design). Consumers valued EBCD's narrative focus and ‘structured’ process, which offered ‘different options for involvement’. Choosing to participate in ‘some or all’ EBCD phases (i.e., phased involvement) was considered particularly suitable to young people and families who described busy schedules and competing priorities. We chose an online setting to protect the study from Covid‐19 restrictions and to support flexibility for participants located in geographically dispersed areas.

### 2.2 Team composition

Our co‐production team included eight academic researchers and three co‐researchers with lived experience of disability. Roles included lead researchers (J.J.C. and R.T.), co‐researchers (F.O.K., M.Y. and H.S.), co‐design convenors (V.P. and J.B.), evaluators (C.I. and M.W.) and senior researcher support (A.S. and N.S.). Lead researchers and co‐researchers formed a five‐person steering group which led decision‐making and project oversight. Convenors, evaluators, senior researchers and external experts offered consultative support with methodological decisions.

### 2.3 Study design

We used Strnadová's six stages of co‐production [31, 34] to guide our participatory research (see Table 1). This paper represents the team's reflective account of co‐production.

**Table 1:** Co‐production methods and meeting schedule.

Co‐production stage	Co‐production processes	Project steering group online activity schedule
Stage 1: Initiating	Team formation	Early engagement via phone calls/social media/online meetings Expression of interest and co‐researcher selection
	Setting project scope and costs	Meeting 1: Introductions and project overview
	Briefing and training	Training 1: Introduction to research integrity and ethics Training 2 (optional): Point of Care Foundation (PoCF) EBCD toolkit [21] Training 3 (optional): PoCF virtual EBCD course (×4 2‐h sessions)
Stage 2: Planning	Deciding on research methods	Meeting 2: Research questions, problem identification and sample frame Meeting 3: Experience gathering: whose story and data collection methods Meeting 4: EBCD evaluation and language choice
	Ethics application preparation	Meeting 5: Participant‐facing documents Meeting 6: Preparing an ethics application
Stage 3: Doing	Supporting recruitment and project administration	Meeting 7: Recruitment and participant selection via sample frame Onboarding of a co‐researcher as project administrator Email correspondence on participant selection
	Preparing for qualitative methods	Meeting 8: Virtual observations of existing cycling services and discussion on reflexivity
Stage 4: Sense‐making	Interpreting qualitative analysis	Meeting 9: Interpreting candidate themes and categorising photographs from photo‐elicitation interviews Meeting 11: Synthesising shared objectives for the final co‐design workshop
	Refining qualitative findings	Meeting 10: Digital story co‐production Meeting 12: Making sense of co‐design
Stage 5: Sharing	Preparing for presentations	Meeting 13: Preparing for the celebration event
	Presentation of findings	Presentation 1: Celebration event with EBCD participants Presentation 2: Community of practice with disability academics Contributing to this journal article
Stage 6: Reflecting	Critical reflection on involvement and impact	Meeting 14: Reflecting on co‐production Meeting 15: Reviewing the co‐production paper together
	Deciding on future steps	Optional meetings on subsequent grant applications

Abbreviations: EBCD, experience‐based co‐design [19]; PoCF, Point of Care Foundation [21].

#### 2.3.1 Study Procedures

Co‐production was conducted through videoconferencing, email and asynchronous document review (e.g., comments and track changes). Phone calls and text messages supplemented communication. Decision‐making was informed by project objectives, discussion, consensus [35] and, where required, voting. Our field notes, attendance log, researcher diaries and meeting minutes formed a co‐production audit trail. Minutes were itemised according to co‐researcher contributions, project leads' contributions and noted decisional flow from proposals to actions. We voted when proposals met consensus, but reservations remained. Votes were cast via online polls (within meetings) or email. Each steering group member held an equal vote, and a majority of three votes was required to carry a proposal.

#### 2.3.2 Stage 1: Initiating

Consumers were invited to express interest in co‐researcher roles through an open advertisement hosted on a REDCap form [36]. We approached known networks, including individuals (*n* = 5), disability organisations (*n* = 3) and research groups (*n* = 1) by phone or email. Applicants received a project overview and position description informed by co‐production guidelines [31]. We considered suitability criteria, time capacity and cycling interest when selecting co‐researchers. After three co‐researchers were onboarded, we consolidated project objectives by discussing external factors (e.g., prospective grants), our EBCD deliverables and project constraints. Project objectives included (i) developing an inclusive EBCD process, (ii) overseeing the EBCD process and (iii) involving co‐researchers across all research stages. We communicated roles and responsibilities through terms of reference (Supplemental File S1— Section A). Co‐researchers participated in introductory training led by J.J.C. and R.T. on research ethics, EBCD and consumer involvement.

#### 2.3.3 Stage 2: Planning

Next, we prepared methods and a protocol for the University Human Research Ethics Committee's review. Before meetings, lead researchers sent preparation material detailing agenda items, meeting goals and preparatory activities (e.g., reflexive exercises and pilot testing). Co‐researchers were paid for 1 h of preparation time per meeting. We pilot‐tested two participant questionnaires from the Public and Patient Engagement Evaluation Tool (PPEET) Version 2.0 [37] (Optional Demographics and Module B Ongoing/Long‐term Initiative), the Patient Engagement In Research Scale (PEIRS) [38] and study‐specific surveys. The PPEET and PEIRS are publicly available surveys used to evaluate or describe engagement in consumer involvement initiatives. Piloting enabled feedback on language, usability, completion time and contextual adaptations. We developed easy‐read information using guidelines [39] and prepared recruitment material to be accessible via multiple sources (e.g., video and easy read).

#### 2.3.4 Stage 3: Doing

Co‐researchers supported recruitment by sharing advertisement material and selecting participants using our sample frame (Supplemental File S1—Section B). One co‐researcher (M.Y.), who had skills in project management, was employed as a project administrator. The administrator scheduled participant activities, sent preparatory material and collected quantitative data (e.g., attendance log). We prepared for qualitative methods by observing local cycling programmes via video archives. Observations gathered our personal assumptions of early cycling opportunities through an EBCD‐informed template.

#### 2.3.5 Stage 4: Sense‐Making

During experience gathering, adapted methods were used to analyse photo‐elicitation interviews. Lead researchers developed candidate themes from transcripts using Steps 1–3 from reflexive thematic analysis [40]. Co‐researchers refined candidate theme wording and offered personalised interpretations of preliminary findings. A three‐part photo‐elicitation interview analysis described photo intentions, interpretations and summated stories from the photo‐text product. Co‐researchers sorted photographs into categories and identified core photograph sets [41, 42]. A digital story [43] triangulated the candidate themes and categorised photographs through a script and video. Digital storytelling was led by a co‐researcher (F.O.K.), who was skilled in video production. Lead researchers developed the script, and F.O.K. produced, edited, annotated and narrated the video. During co‐design, we synthesised updates from convenors and suggested priority objectives (e.g., intervention elements [4]) for the combined workshop. Before the celebration event, we discussed the convenors' co‐design report, our methods and study limitations.

#### 2.3.6 Stage 5: Sharing

Sharing involved preparing visual presentations, presenting findings and contributing to this article. We decided on the celebration event's format, roles, invitation and an event gift. Each member of the steering group prepared slides and presented findings. ‘Meet the researcher’ videos and an infographic were prepared as knowledge translation outputs.

#### 2.3.7 Stage 6: Reflecting

Reflections discussed the level of involvement, impact and future directions for co‐production research. We used involvement matrices [26, 44] to describe the perceived level of involvement at each co‐production stage. An impact taxonomy [32] aided descriptions of co‐researcher impact. We reported barriers, facilitators and limitations in the GRIPP2‐SF [1] (Supplemental File S1—Section A, Table S1).

#### 2.3.8 Ethical Considerations

While completed in an ethical manner, this part of the research did not require formal ethical approval as research data was not collected. Consumers from our early engagement and co‐researchers granted permission to use quotes and findings gathered during co‐production.

## 3 Results: Outcomes of Co‐Production

Our results are presented in three sections: (1) EBCD adaptations; (2) our primary co‐production output—the CycLink Co‐design Study protocol and (3) co‐researcher involvement and impact in co‐production.

### 3.1 Adaptations to EBCD

We adapted EBCD to our population's diverse support needs by tailoring methods for inclusivity, intervention design and the online setting (see Table 2). Adaptations sought to facilitate experience sharing, choice‐making and engagement of all co‐designers. We chose design principles rather than an entire complex intervention as a feasible EBCD deliverable for this development phase. Setting a desired deliverable early enabled us to plan around our constraints of time frames, convenor availability and funding. We retained EBCD's phases and steps, but moved through an accelerated process, whereby workshops were shorter and conducted over 8 weeks. Design activities focused on early cycling experiences and intervention elements. We aimed to enhance engagement through preparation materials, a digital display tool and breakout rooms. Shorter workshop durations (i.e., 1–2 h) were considered more practical for retaining online engagement. Delivering EBCD online required several ethical safeguards. For example, to protect online safety, we used a study‐specific email address, trained participants in photo‐taking, agreed‐upon expected etiquette and separated adults and children into different workshops. We screened participants' online capabilities by phone/survey (e.g., ability to use email, surveys and videoconferencing), promoted use of a familiar device (e.g., personal iPad), remained on one website during videoconferencing and offered technical support (e.g., tip‐sheet) as required.

**Table 2:** Adaptations to the EBCD process.

Phases	Traditional EBCD steps [21]	Adapted EBCD for CycLink Co‐Design Study
Setting up	**Project steering group formation** Focus: Quality improvement in service delivery or care pathway Preparation: Seek organisational support for co‐design (leadership and budget) Team roles/composition: Project lead, qualitative researcher, steering group, psychosocial support (e.g., counsellor) and facilitator Outcomes: Time frames and project infrastructure (e.g., facilities)	**Initiating and planning for research** Focus: Develop a community cycling intervention with young people with disability, parents and community partners Preparation: Early consumer involvement and consultative advice. Secure grant funding and prepare study protocol Team roles/composition: Lead researchers, co‐researchers, project steering group, academic researchers, convenors and evaluators Outcomes: Feasible EBCD deliverable (i.e., programme principles), objectives, time frames, adapted process, online infrastructure (e.g., well‐being resource; engagement through (i) email, (ii) survey tool and/or (iii) videoconference) and ethics application
Experience gathering	**Step 1: Site‐based observations** Format: In‐person visit to a single site (e.g., ward) by the project lead Outcome: Findings on routine events and experiences shared with staff in Step 5	**Step 1: Virtual observations** Format: Guided reflection of 3 local cycling services by the steering group using videos and an EBCD‐informed observation template Outcome: List of local disability cycling services shared with convenors. The steering group developed reflexivity skills
	**Step 2: Staff interviews** Recruitment: Staff identified by key contacts and briefed on the project Preparation: The interview guide is often shared with participants Method: Semi‐structured interviews conducted in‐person or by phone, OR selected from national archive [45]	**Step 2: Photo‐elicitation interviews with community partners** Recruitment: Consent and participant characteristics survey were completed. Steering group selected participants via a sample frame Preparation: Photo‐interview tip‐sheet ± worked example Method: Photo‐elicitation interviews (see Section 3.2.2) lasting 30–45 min conducted online with lead researchers
	**Step 3: Patient and family interviews** Recruitment: Patients were identified by staff and briefed on the project and provided consent Preparation: Interview guide is often shared with participants Method: Semi‐structured interviews lasting 60–120 min; interview typically conducted in person and video‐recorded	**Step 3: Photo‐elicitation interviews with young people and parents** Recruitment: As per community partners (above) Preparation: Photo‐interview tip‐sheet ± worked example. Adjustments (on request):* file folder [46] ± Polaroid camera ± assistive technology (e.g., camera‐mount and adapted switch) and props (e.g., coach's whistle and detective's magnifying glass). Method: Photo‐elicitation interviews (see Section 3.2.2) lasting 30‐45 min conducted online with lead researchers ± familiar communication partner. Multimodal communication [47] and dyad perspectives were recorded
	**Step 4: Videos edited, development of touchpoints and trigger film** Method: Qualitative analysis used to develop touchpoints in the patient experience journey Outcome: Interview films edited to short clips and total trigger film lasts approximately 20–30 min	**Step 4: Co‐production of a digital story** Method: Steps 1–3 of rTA [40] and photo‐elicitation interview analysis [41, 42] completed by lead researchers Process: (a) Co‐researchers refined candidate themes and categorised photographs; (b) lead researchers developed a strength‐based script by triangulating findings; (c) co‐researcher produced, edited, annotated and narrated the digital story; (d) co‐design convenors reviewed and contributed to further iterations Outcome: Final 4‐min digital story video co‐produced by steering group (including subtitled version with audio captioning)
Co‐design	**Step 5: Staff feedback event** Method: Facilitated 2‐h face‐to‐face meeting held with staff and leadership Format: Structured agenda, sharing of observations and interview findings Outcome: identification of key improvements (staff perspective)	**Step 5: Co‐design workshop with community partners** Method: Facilitated 90‐min online workshop. Digital display tool gathered collaborative input on current cycling programmes Format: Structured agenda, sharing of observation findings and further identification of early cycling opportunities Outcome: Beginner cycling journey map and consolidation of touchpoints for improvement (community partner perspectives)
	**Step 6: Patient feedback event** Method: Facilitated (approximately) 2‐h face‐to‐face meeting Format: Structured agenda including sharing the trigger film, discussion on stages in the patient's journey (e.g., via emotional mapping) and key touchpoints Outcome: Identify priority improvements (patient perspective)	**Step 6: Co‐design workshop with lived experience group (young people aged > 18 years, parents and carers)** Method: Facilitated 90‐min online workshop. Digital display tool gathered collaborative input on past experiences and ‘an ideal’ CycLink programme Format: Structured agenda, creative methods (e.g., comic‐strip storyboard and journey mapping with colour and symbols) Outcome: Emotive journey map and consolidation of touchpoints for improvement (lived experience perspectives)
	**Step 7: Joint workshop** Method: Facilitated 3‐h face‐to‐face meeting held with staff and leadership Format: Structured agenda; trigger film is re‐shared with the whole group, followed by group discussion and breakout groups Outcome: Consensus on Specific, Measurable, Attainable, Realistic and Timed (SMART) goals and ‘target areas for improvement’	**Step 7: Combined workshop with lived experience group and community partners** Method: Facilitated 2‐h online workshop with a mixed group. Digital display tool gathered collaborative input on principles. Format: Structured agenda (input from steering group), digital story shared with whole group and prioritisation exercises for CycLink's intervention items [4] via early CycLink prototypes. Voting on prioritisation exercises by counting emoticon responses Outcome: Consensus on design principles of CycLink programme
	**Step 8: Small co‐design teams** Formation of several smaller groups (mixed composition of parents and staff) based on different priority areas Pragmatic step of developing ‘ideas into actions’, consensus‐building on service improvements and changes	**Step 8: Children's advisory group (aged 8–15 years)** Methods: Two facilitated 45–60‐min online workshops conducted after Steps 6 and 7. Preparation material supplied. Format: Workshops structured into 4 parts: (i) introduction, (ii) chatting (e.g., icebreakers), (iii) sharing (e.g., open/close‐ended prompts) and (iv) choosing (e.g., voting). Adjustments included: ‘first and then’ instructions, pairing language with Pictorial Communication Symbols (PCS®), categorising ideas (easy vs. difficult) and voting via gesture (nod, shake or shrug). Facilitation strategies included digital display tool, role‐modelling (worked example and turn‐taking), Hanen Centre's SPARK™ communication strategies (e.g., wait and actively listen) [48] and parent involvement (e.g., rephrase and clarify) Outcome: Synthesis of children's ideas and adult priorities
Celebration	**Step 9: Celebration event** Feedback event approximately 6–9 months after the joint workshop	**Step 9: Celebration event** Online presentation 3 months after the joint workshop

Abbreviations: AEBCD = accelerated experience‐based co‐design, EBCD = experience‐based co‐design, rTA = reflexive thematic analysis, SMART = Specific, Measurable, Attainable, Realistic and Timed.

* File folder: This adapted and semi‐structured method [46] helps scaffold photo‐taking for people with intellectual disability. The method involves posting the participant a Polaroid camera and four card envelopes, which are pre‐labelled with suggested themes.

### 3.2 The Outputs of Co‐Production: The CycLink Co‐Design Study Protocol

We developed a two‐part study to *use* (Part 1) and *evaluate* (Part 2) co‐design. We received ethics approval from the Human Research Ethics Committee at the University of Melbourne (reference: 22588) and collected data from July 2022 to April 2023. We aimed to recruit young people, parents and community partners via targeted advertisement. Potential participants accessed the study via an expression of interest before progressing to consent procedures. Inclusion criteria sought young people with disability aged 8–30 years with: (i) cycling goal(s); (ii) recent experiences of learning to cycle; (iii) residence in Victoria, Australia; and (iv) intentional communication (reliable yes/no response and ability to express opinions) in English. Eligible parents and community partners needed to have experience supporting young people with disability to cycle.

#### 3.2.1 Ethical Procedures

Three consent pathways were developed to accommodate three distinct study populations: (i) adults without intellectual disability; (ii) children and young people aged < 18 years and (iii) young people aged > 18 years with intellectual disability or complex communication needs. Adults without intellectual disability were invited to provide informed consent via an online consent form. We sought parental consent, alongside the young person's consent, for children and adolescents aged < 18 years through a similar online form. Young people with intellectual disability or complex communication needs were invited to provide verbal consent with a guardian or familiar communication partner by videoconference. The verbal consent process followed similar steps to those described by Arscott et al. [49]. We modified this process to screen‐share easy‐read information and consent options via videoconferencing (Supplemental File S1—Section B). The first step identified how a young person communicated a reliable yes or no response. Next, we shared the easy‐read information. Then, we screened their understanding of the study via a series of yes/no questions. The final step allowed young people who understood the study's nature, risks and benefits to provide verbal consent. Supported decision‐making strategies were used with young adults who could not provide verbal consent but wanted to participate. In these instances, assenting young people needed third‐party consent from an appointed individual (e.g., guardian, advocate or supportive attorney) and could nominate a preferred communication partner for dyad responses. Communication partners were briefed on strategies to maximise young persons' participation in sharing perspectives (Supplemental File S1—Section B). In cases where assent was not possible, parents/carers could participate as ‘experts by experience’ offering proxy perspectives [50].

As consent is an ongoing process, we reminded participants of the voluntary nature of the research before each activity. During workshops, one convenor observed for behavioural cues that may indicate dissent, refusal or withdrawal. Behaviours included actions (e.g., repetitive yawning) or communication strategies (e.g., expressing desire to leave or looking to exit points). Where dissent/withdrawal or coercion (e.g., from a support person) was suspected, researchers arranged a follow‐up call or breakout room to collaborate on a participation plan. Planning for subsequent activities considered amended roles, additional supports and/or reassessing ongoing involvement (e.g., withdrawal).

All participants had opportunities to debrief by phone and access a resource on ‘minding your wellbeing’ during co‐design. Participants were reimbursed with a $25 AUD voucher per research activity lasting ≤ 60 min for both parts of the study.

#### 3.2.2 Part 1: Use of Co‐Design

**Figure 1:**
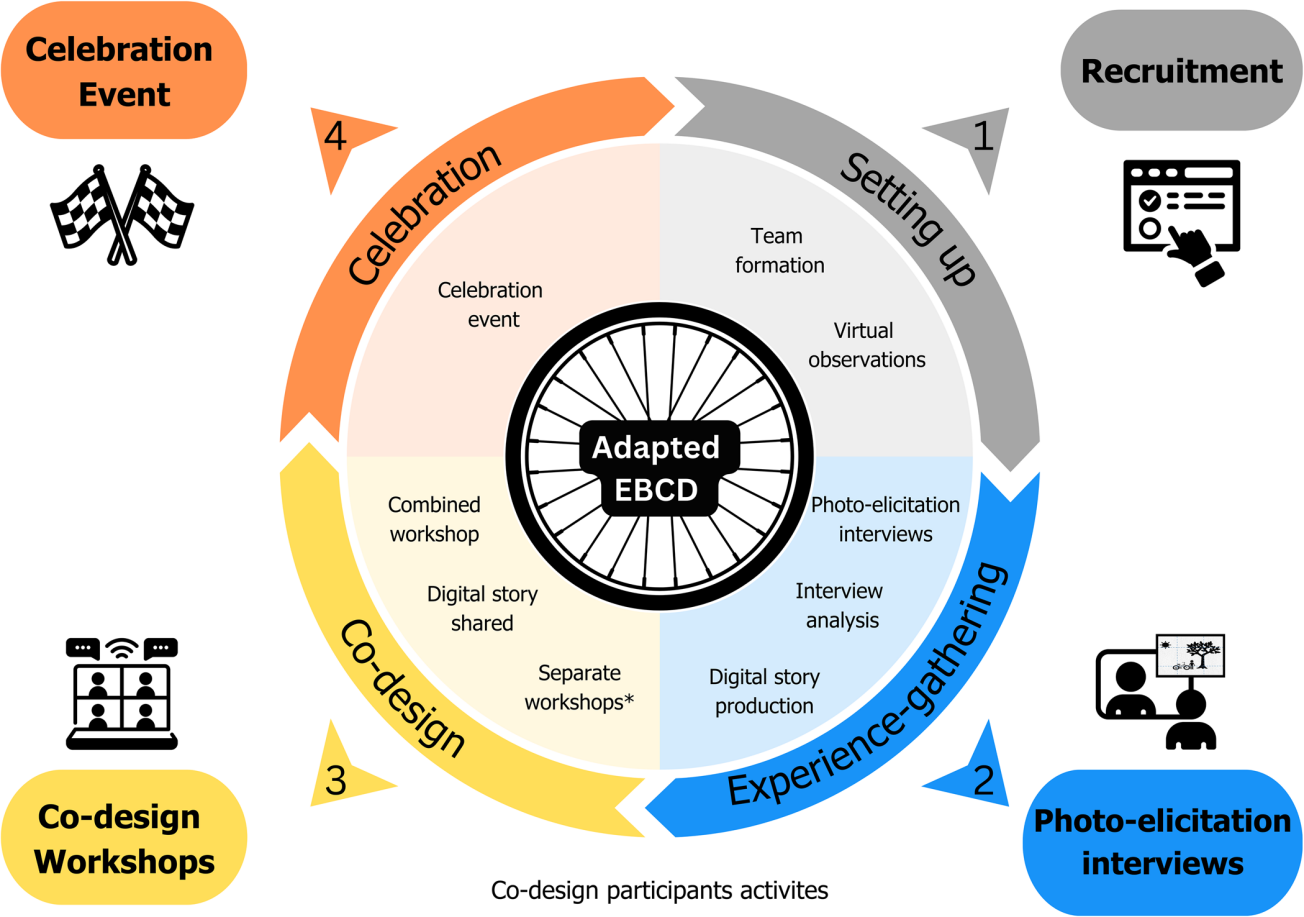
The CycLink Co‐design Study's adapted EBCD process. Notes: Co‐designers interacted with the research activities in the outer perimeter. EBCD phases (dark colours) and steps (light colours) are represented in the centre of the figure. *Separate workshops included a community partner workshop, lived experience group workshop and two children's advisory panel workshops.

Part 1 used multiple qualitative methods guided by our four‐phase EBCD process (see Figure 1). Colour and visuals oriented participants to the process. All participants were invited to complete a ‘characteristics survey’ to describe demographics, identify support needs and aid selection for research activities through maximum variation sampling (Supplemental File S1—Section B).

##### 3.2.2.1 Experience‐Gathering Phase: Photo‐Elicitation Interviews

3.2.2.1.1

*Participants*. Experience gathering aimed to invite at least 15 participants (8 young people, 2 parents and 5 community providers) to photo‐elicitation interviews. *Data collection*. Photo‐elicitation interviews asked participants to provide three photographs of early cycling experiences that explored barriers/facilitators to community cycling. Participants could take new photographs or choose pre‐existing photos. Before interviews, participants received a ‘photo‐tip sheet’ (Supplemental File S1—Section B), guiding them on ethical considerations for photo‐taking, such as asking for consent and including recognisable features. A QR code or weblink enabled participants to obtain third‐party consent from people who featured in photographs. Photographs were used as conversation‐starters during photo‐elicitation interviews and complemented our semi‐structured interview guide. *Data analysis*. We used adapted methods (see Methods—‘Sense‐Making’) to develop a script and co‐produce a digital story.

##### 3.2.2.2 Co‐Design Phase: Workshops

The co‐design phase involved a total of five online workshops for adults and children. *Participants*. We aimed to recruit 16 adult participants and up to 10 children to attend two workshops each. *Data collection*. Both adults' and children's workshops included icebreakers, online agreements, structured agendas and regular breaks. Adult workshops were facilitated by two experienced convenors (V.P. and J.B.) who developed co‐design activities iteratively based on co‐designers' needs and study objectives [51]. A digital display tool was used to present activities and collate co‐designers' inputs via sticky notes. Separate adult workshops were held for community partners and people with lived experience. These workshops were followed by a combined workshop bringing the two adult groups together. Researchers J.J.C. and R.T. facilitated two separate children's workshops with 8–15‐year‐olds. Children's workshops were informed by adult workshops and aimed to consolidate emerging ideas from co‐design. Creative methods, small group sizes and adjustments (see Table 2) helped facilitate children's participation. *Data analysis*. Convenors synthesised key findings into conceptual designs to illustrate experiential goals and component parts, informing the CycLink's principles. EBCD outcomes will be published separately.

##### 3.2.2.3 Celebration Phase: Online Presentation

All consenting participants were invited to attend an online celebration event where the study's findings were presented.

#### 3.2.3 Part 2: Embedded Process Evaluation of the CycLink Co‐Design Study

**Figure 2:**
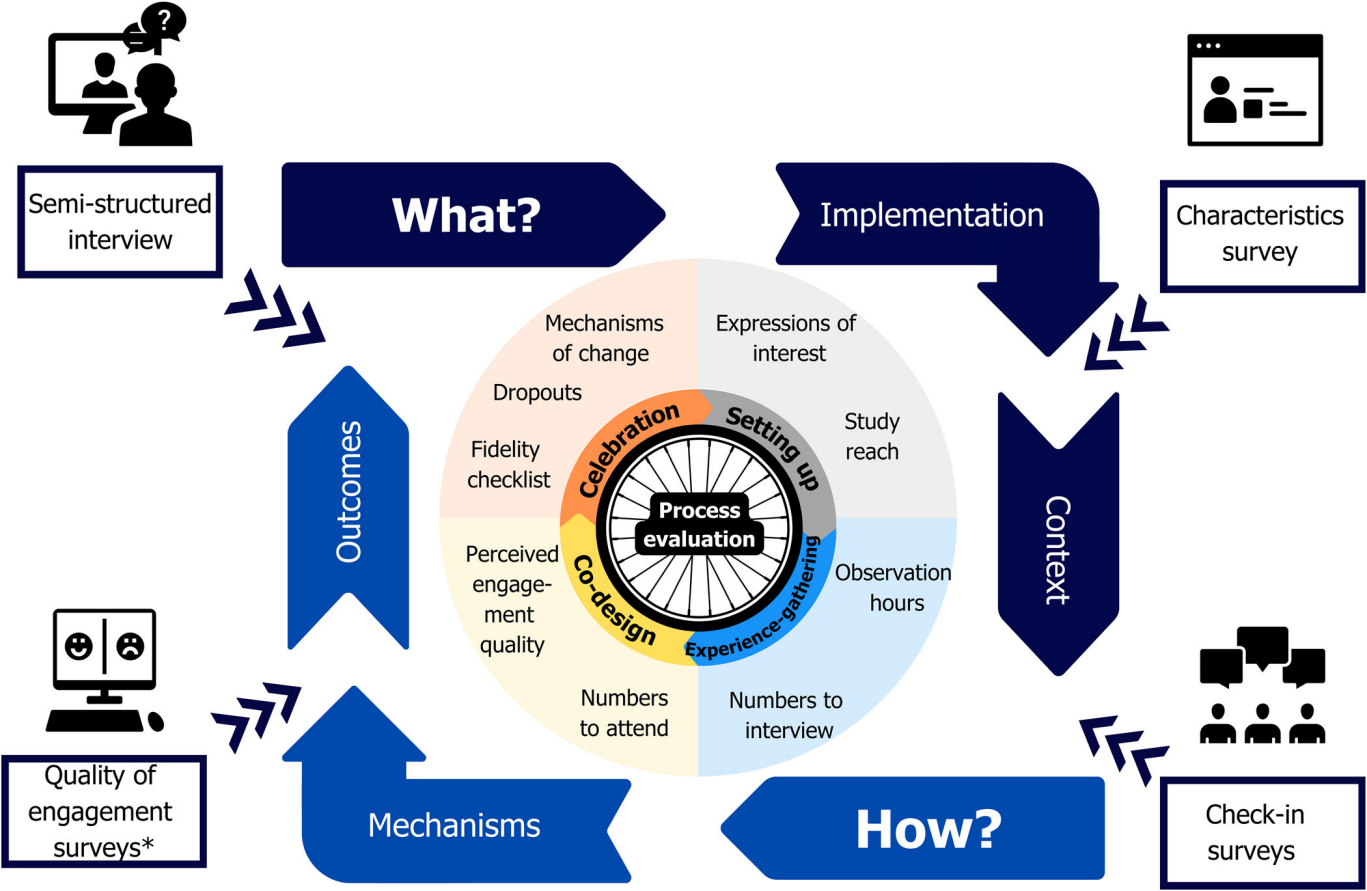
Our adapted EBCD process evaluation. *Note:* *Quality of engagement surveys included the PPEET and young person evaluation surveys (study specific).

We co‐produced a process evaluation of our EBCD (see Figure 2) involving a convergent parallel mixed methods design [52]. *Participants*. All participants from Part 1 were eligible to participate. *Data collection* was embedded into co‐design methods (e.g., attendance records and check‐in surveys) and gathered after the final workshops (e.g., questionnaires or interviews). The PPEET (Module B Long term/Ongoing Initiative Questionnaire), a study‐specific young person's evaluation questionnaire, routine data (e.g., workshop invitation/attendance) and an EBCD [20] fidelity checklist formed the quantitative dataset. Our young person's evaluation questionnaire used subsections of the PPEET [37] and included topic sentences that reflected concrete events, tasks or people within the study. Consultative advice from speech and language pathologists identified visual rating scales aligned to Talking Mats™ [53] (e.g., ‘Yes/No/Unsure’ and ‘Like/Don't like/Unsure’) and corresponding Pictorial Communication Symbols®. The fidelity checklist rated adherence (completed vs. uncompleted/unclear) to 10 improvement activities identified by Green et al. from the PoCF toolkit [20]. Qualitative data included workshop recordings, open‐ended surveys (factors to stop, start and continue during co‐design) and semi‐structured interviews. An evaluation team, independent of the co‐design process, emailed surveys, conducted semi‐structured interviews and audited the fidelity check. *Data analysis*. We plan to analyse qualitative and quantitative datasets separately, before merging the findings to gain a deeper understanding of engagement quality and co‐design mechanisms [54]. Evaluation findings will be published in a subsequent paper.

### 3.3 Level and Impact of Co‐Researcher Involvement in Co‐Production

#### 3.3.1 Co‐Researchers Engaged

Three co‐researchers joined our project steering group and met 18 times over 2 years. All were new to co‐researcher roles, of Caucasian ethnicity and had concurrent commitments (e.g., employed or studying full‐time).

#### 3.3.2 Participation Attendance and Involvement in the Process

Steering group attendance was high (see Table 3). One co‐researcher was unable to attend several joint meetings due to clashes with work (*n* = 5) and health appointments (*n* = 1). Follow‐up phone calls (*n* = 5) accommodated their updates and contributions.

**Table 3:** Steering group involvement throughout co‐production.

Co‐production stage	Online engagement	Co‐researcher attendance (*n/N*)	Lead researcher attendance (*n/N*)	Time^a^ (h)
Initiating	EOI^b^ process	3/3	2/2	0.6
	Meeting 1	3/3	2/2	1.0
	Training 1	3/3	2/2	2.0
	Training 2	0/3	2/2	5.0
	Training 3	0/3	1/2	8.0
Planning	Meeting 2	3/3	2/2	1.1
	Meeting 3	2/3	2/2	1.0
	Meeting 4	3/3	2/2	1.1
	Meeting 5	3/3	2/2	1.1
	Meeting 6	2/3	2/2	1.1
Doing	Administration^c^	1/1	2/2	30.0
	Meeting 7	2/3	2/2	1.1
	Meeting 8	3/3	2/2	1.1
Sense‐making	Meeting 9	3/3	2/2	1.1
	Meeting 10^d^	1/1	1/1	30.0
	Meeting 11	2/3	2/2	1.2
	Meeting 12	3/3	2/2	1.2
Sharing	Meeting 13	3/3	2/2	1.1
	Presentation 1	3/3	2/2	1.3
	Presentation 2^e^	1/1	2/2	0.5
Reflecting	Meeting 14	2/3	2/2	1.0
	Meeting 15	2/3	1/2	1.1

*Note:* Attendance recorded as *n/N*, where *n* = number attended, *N* = total invited.

^a^ Time spent attending online meeting, note this figure does not include the 60 min of preparation time before most meetings.

^b^ EOI = Expression of Interest.

^c^ Administration: individual co‐researcher involvement over several months.

^d^ Individual co‐researcher activity relating to digital story production.

^e^ Individual co‐researcher representation during the community of practice presentation.

**Figure 3:**
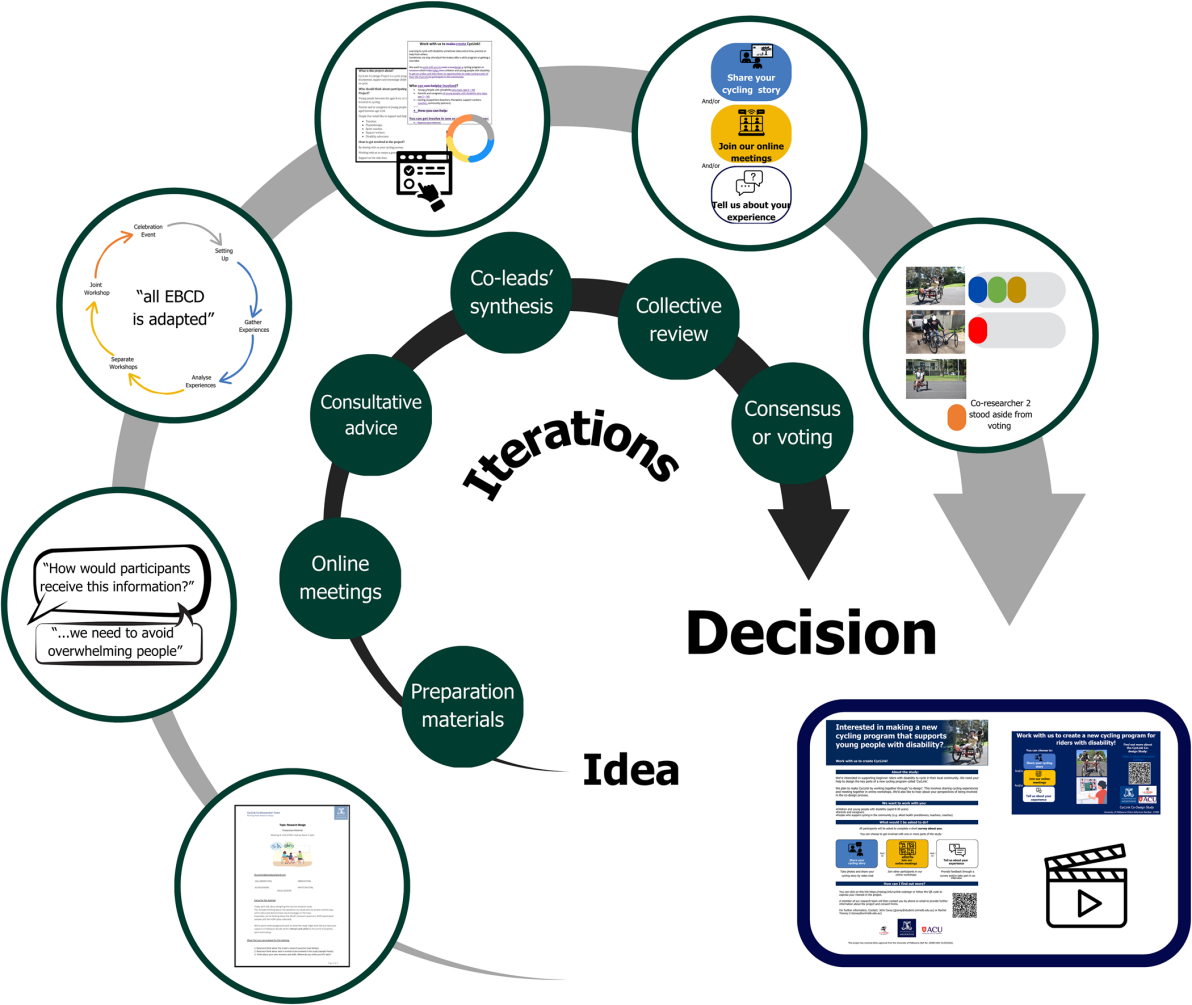
Iterative decision‐making process example: developing our recruitment material. *Note:* The inner dark arrow represents our decision‐making process. The outer light arrow illustrates examples of an agenda (Box 1), co‐researchers' discussion points (Box 2), convenor recommendations (Box 3), conceptualising materials (e.g., proposed wording, colour and symbols) (Box 4), reviewing proposals and simplified graphics (Box 5), voting on preferred imagery (Box 6) and the final recruitment materials (Box 7).

Planning and sense‐making stages were our most time‐intensive periods. Decision‐making was iterative and co‐researchers were influential in reducing jargon and enhancing the clarity of proposals (see Figure 3, Supplemental File S1—Section A, and Figures S1 and S2).

**Figure 4:**
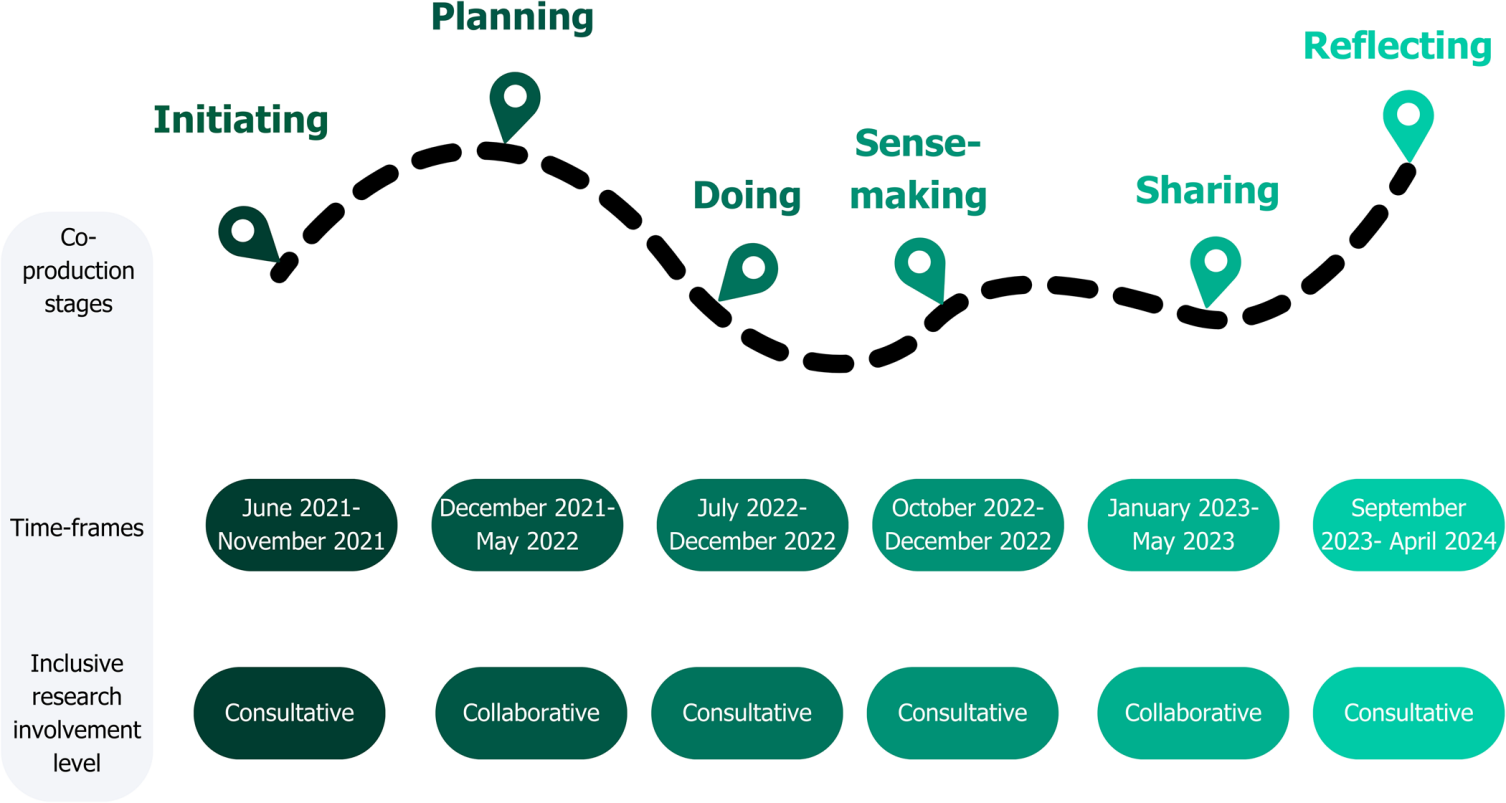
Ebb and flow of co‐researcher involvement.

Figure 4 represents the ‘ebb and flow’ of co‐researchers' involvement throughout co‐production. Collaborative involvement was most evident for choosing methods, adapting materials and developing the digital story. Co‐researchers felt empowered to offer ‘unsolicited advice’ and be equal decision‐makers. However, at times they felt ‘sidelined’ in leading research activities and wanted greater involvement in collecting ‘cycling‐related’ data. Ultimately, co‐researchers valued flexibility, role clarity and adequate time to meaningfully participate in co‐production.

#### 3.3.3 Impact

The impacts of co‐production are described in Table 4. Our co‐production resulted in an inclusive and safe online process that could empower co‐designers with diverse abilities to participate. Co‐researchers reflected on a ‘person‐centred’ impact, illustrated through simplified involvement options, accessible materials and relatable knowledge translation outputs. They strengthened the relevance and trustworthiness of the study's findings by modifying wording and challenging academic researchers' assumptions. Co‐researchers enjoyed sharing cycling expertise and valued developing knowledge and skills in research. Personal successes resulted in one co‐researcher securing ongoing employment in academia and another writing a cycling blog.

**Table 4:** Impact of co‐production on project outcomes and outputs.

Co‐production stage	Consumer and co‐researcher contributions (elements/activities)	Evidence to support decisions and impact (e.g., research team quotes) (metric/evidence)
Stage 1: Initiating	Two consumers initiated contact with researchers on cycling research Identified two consumer involvement roles (i.e., co‐researchers and co‐designers) Contributed to the grant application Expressed interest via form Validated researcher‐identified evidence gaps Endorsed the whole team's agreement on project scope/objectives Endorsed convenors' advice to ‘design principles’ as EBCD deliverable Engaged in formal training on ethics Engaged in ‘on the job’ training (reflexivity, critical discussion, experiential learning and scaffolded materials) Identified opportunities through the terms of reference document (e.g., develop a digital story)	Expression of interest attracted 11 applicants: *n* = 7 identified as living with disability *n* = 4 identified as a parent to a child with disability Three co‐researchers joined the team as casual research assistants ($45–50 AUD/h): *n/N* = 2/5 consumers from early engagement joined as co‐researchers *n* = 1 consumer new to the research team Representation of people with diverse lived experiences of cycling with disability: Ages: 18, 47 and 50 years Disability: cerebral palsy (*n* = 2), developmental/genetic condition (*n* = 1) Cycling experiences: Learned to cycle as a child versus as an adult versus exploring options Cycling equipment use: eTrike versus adapted trike versus balance bike versus 2‐wheeler Education: Some post‐secondary school (*n* = 1) and university qualification (*n* = 2) Re‐purposed existing grant for reimbursement of both co‐researchers and co‐designers
		Identified phased involvement as a pragmatic way for young people/families to participate
		Endorsed EBCD as a method that centralises phased involvement and storytelling: ‘Experiences are key … EBCD offers us a blueprint’
Stage 2: Planning	Re‐framed research question and study design to include ‘EBCD’ Developed a sample frame or ‘participant wish list’ to guide maximum purposive sampling Endorsed photo‐elicitation interviews as a suitable creative method Developed topic guide questions and prompts for photo‐elicitation interviews Shared expertise from other life and project roles (e.g., photo‐consent) Pilot‐tested PPEET's demographics module^a^, evaluation module B^b^, PEIRS survey and study‐specific surveys Voted on recruitment material, sample frame and evaluation surveys Proposed evaluation time points Supported choice of language, pictorial symbols and accessible font Wrote personal summaries for ethics application Developed first drafts for (i) recruitment material, (ii) photo‐elicitation interview guide and (iii) well‐being resource Reviewed all participant‐facing documents Pre‐tested the study's online infrastructure and participant pathway using mobile and computer devices	Identified three phased involvement options: (1) photo‐elicitation interview, (2) co‐design workshops and (3) evaluation survey and/or interview.
		Used colour, visuals and ‘clear expectations’ (i.e., time/task demand) to illustrate options
		Included ‘all disabilities’ in eligibility criteria: ‘broad is best … cycling shouldn't be limited by your diagnosis’. Emphasised recruiting diverse experiences over specific diagnoses
		Identified preferred terminology for project materials and surveys (e.g., PPEET): ‘…“patient” makes it sound like I am unwell and sick … it should be people with disability.’
		Emphasised participation (e.g., factors that ‘challenge’/enable) and learning journey as foci of photo‐elicitation interviews
		Decided that all co‐designers should be invited to evaluate EBCD either informally (e.g., via check‐in survey) or formally (e.g., as Part 2 participants). Embedded check‐in surveys were perceived as useful: ‘they give us the chance to change course if we need.’
		Offered feedback on the PPEET's demographics module^a^: Average time to complete (*n/N* = 3/3): 1.8 min Adaptations for the Australian context (e.g., schooling and Indigenous populations) Offered feedback on the PPEET's evaluation module B^b^: Average time to complete (*n/N* = 3/3): 7.0 min Usability/adaptations: preference to be written in past tense (‘it's less confusing’) and replace ‘organisation’ with ‘researchers’ to reflect research context Identified photo‐consent pathway for non‐participants who featured in a participant's photo
		‘I'm interested but…’: The decision was made not to be involved as both co‐researchers and co‐design participants. The team discussed time burden, role commitments (i.e., already involved in dual roles) and power imbalance (e.g., in analysis) as important factors
Stage 3: Doing	Shared recruitment material with personal networks Selected EBCD participants Collected recruitment data Identified recruitment gaps (e.g., fathers and CALD community) Collected workshop attendance/non‐attendance data Checked survey completeness and triggered participant reminder emails Undertook virtual observations and reflected on qualitative positionality Collated slide deck for photo‐elicitation interview analysis (i.e., photos/text) Developed convenor prompts for children's co‐design workshops	Shared recruitment material with *n* = 4 organisation leads and *n* = 2 community of practice groups using personal networks
		Populated the recruitment flow chart and attendance log with prospective quantitative data
		Targeted advertisement led to *n* = 1 father joining the study as a co‐designer following the identification of a recruitment gap
		Co‐researchers asked ‘what programs or resources are already out there?’ and ‘who's missing out?’ This led to a list of local cycling opportunities being shared with co‐designers
		Reflected on the ‘different hats’ (i.e., perspectives) worn in qualitative analysis and identified a positionality that: Generated pragmatic knowledge and sought practical solutions/ideas Valued experiential knowledge Differentiated personal lived experiences from co‐designers' perspectives
Stage 4: Sense‐making	Interpreted the lead researchers' preliminary analysis Refined wording of candidate themes Discussed photograph intentions and interpretations Categorised photographs into core sets Synthesised convenor updates and identified objectives/design activities (e.g., voting) for the combined workshop Offered insights on the relevance and relatedness of the final co‐design report	One co‐researcher related strongly to the candidate themes developed by lead researchers: ‘As a parent of a child at the pre‐biker phase … the [candidate] themes address all the issues I've come up against’
		Modified a candidate theme from ‘family helps keep the wheels in motion’ to ‘supporters help keep the wheels in motion’ to be cognisant of young adults who accessed support from allied health professionals or coaches
		Led the co‐production of the digital story: ‘We need to create a succinct narrative and core script … 250 words at the most … needs to highlight the strengths and the issues’
		Emphasised meaningful and relatable outcomes that could be used readily by riders with disability and their families
	Re‐directed the team back to pertinent findings for knowledge translation Developed own slides for presentation Co‐produced slide deck for a celebration event Collaborated on an infographic summarising the study's findings	Emphasised further sharing of the digital story: ‘It offers a clear way to communicate’
Stage 5: Sharing		All (*n/N* = 3/3) co‐researchers presented content at the celebration event
		Advocated sharing local opportunities/practical implications, in addition to study findings: ‘What now? … some parents came in [to co‐design] wanting something for their family or child … need to share principles … but also social media stuff on local cycling…’
Stage 6: Reflecting	Collaborated on research paper and development of the GRIPP2‐SF (Supplemental File —Section A, Table S1) Discussed the CycLink project's next steps Identified opportunities for future co‐researcher involvement in the CycLink project and broader disability research Reflected on personal outcomes and learning	Supported the development of Figure 4: ‘the dotted line shows it wasn't a clear rigid path… aspect of learning as you go … we had to carve our path … it was iterative’
		Developed an ongoing commitment to research: *n* = *2* co‐researchers named as associate investigators on the external grant application Additional funding secured for co‐researchers to co‐analyse Part 2 (evaluation) *n* = *1* co‐researcher joined another research project and reflected that co‐production ‘prepared [me] for the ride of research’ Reflected on learnings for broader disability or co‐production research: Allocate time, resources and budget for ‘substantive roles’ Retain connection to core motivator(s): ‘We all got involved because we're interested in cycling … but being involved as co‐researchers meant we couldn't get involved in the actual co‐design’ Train skills in qualitative data collection/analysis (e.g., interviews and workshops)

*Note:* We adapted the PPEET's Optional Demographics Questions (Participant Questionnaire) ^a^and Module B: Ongoing/Long‐Term Engagement Initiative ^b^for our context.

Abbreviations: AUD, Australian dollar; CALD, culturally and linguistically diverse; EBCD, experience‐based co‐design [19]; GRIPP2‐SF, Guidance for Reporting Involvement of Patients and the Public Version 2 Short Form [1]; PEIRS, Patient Engagement in Research Scale [37]; PPEET, Public and Patient Engagement Evaluation Tool (Version 2) [37].

## 4 Discussion

This reflective account describes how co‐production benefited the planning, conduct and reporting of adapted EBCD. We reflected that EBCD [19, 21] offered us a flexible structure to anchor our co‐design study, but adaptations were essential to include young people with disability. Co‐production [31] harnessed our collective expertise in disability, enabling us to anticipate, plan and prepare for meaningful participation by diverse co‐designers. Choice, access and creativity shaped our adaptations. Co‐production required additional time (8 months) and resourcing, but enabled us to develop an ethically approved protocol and invite under‐represented groups to co‐design. During EBCD, co‐researchers positively influenced the quality of findings and digital storytelling by contributing lived experience interpretations into a fast‐tracked EBCD process. Here, we discuss considerations for inclusive co‐design and critically reflect on co‐researchers' involvement throughout our co‐production.

Similar to studies that have used [50, 55, 56], or planned to use [57, 58] EBCD with people with disability, we found that consumer‐led steering groups and established disability approaches supported inclusivity. Like Pozniak et al. [59], we valued the strength‐based approach [28] for its emphasis on individuals' interests, capabilities and future goals, over deficits and problems. Combining co‐researcher expertise with disability approaches was illustrated through the subtle re‐framing of terminology within evaluation tools and EBCD. We chose terms like ‘people with disability’ rather than ‘patient’ and ‘digital story’ rather than ‘trigger film’. By making these changes, we aimed to retain the emotive foundations of EBCD but avoid tragic or heroic portrayals that can encapsulate ableist views of disability [60]. This was particularly relevant to the digital story, which required several iterations to develop a strength‐based script and video.

We found existing toolkits [27, 61] and guidelines [31, 34, 39] useful for adapting our methods and supporting person‐based and environmental needs. Like Pickering [62], we anticipated that young people's perspectives would be enriched by gathering multiple creative data sources and layering them with parents' and professionals' perspectives. Consequently, photo‐elicitation interviews were chosen over more common experience‐gathering methods like semi‐structured interviews or surveys [20]. Photo‐elicitation interviews can enhance young people's ability to share perspectives and develop rapport [63], and we found the method was readily adapted for online use. Other co‐design studies [55, 56] have demonstrated the need for visual methods and flexibility in workshops. Working online enabled us to readily share visually engaging material (e.g., photographs and Pictorial Communication Symbols®) via digital display tools and video. In workshops, co‐designers could participate flexibly via open discussion, sticky notes and the videoconference's ‘chat’ function. Tailored communication strategies [48, 64] and iterative changes enhanced our preparation materials and facilitation skills based on support needs. Other groups [55, 56] have facilitated workshops using topic cards and Talking Mats™, respectively, which we could have explored with further training. Inviting guest speakers has also sparked workshop discussion [55]. Guest speaker roles could have supported the meaningful involvement of our co‐researchers during workshops. Identifying potential mechanisms on ‘how’ our adjustments impacted participation will be explored in subsequent publications.

Like other researchers [65, 66, 67], we faced an intrinsic tension resulting from our co‐production's academic‐initiated source. A power imbalance resulted as the desired deliverables, time frames, funding, grant stipulations and accountability ultimately sat with the lead researchers. We addressed power imbalances by having a higher representation of co‐researchers on the steering group and attaining consensus on methodological decisions. We ‘handed over power’ by sharing defined roles, illustrating the movement of ideas/proposals/decisions and fostering a collaborative environment which valued experiential knowledge equally.

We found time frames and funding constraints exacerbated power imbalances by compressing opportunities for collaborative decision‐making and involvement. Our time pressures related to co‐researchers' availability for involvement, the complexity of preparing creative participatory methods, the rapid turnaround time between EBCD phases and external deadlines. Our strategies to address these challenges included flexible meeting times (e.g., lunchtime/weekend), resource reallocation (e.g., additional time/budget for digital story production), adapted methods (e.g., triangulating analytic products) and timeline extensions (e.g., 12‐month extension for external grant). The impact of time pressures meant co‐researchers sometimes appeased the team, ‘doing what's most needed and most useful’, and lead researchers sought consultative inputs (e.g., review documents or interpretations) rather than collaborative contributions. For example, we sought feedback on pre‐analysed candidate themes rather than empowering co‐researchers to undertake familiarisation and/or coding. This decision was based on our need for a rapid translation of experience‐gathering findings to digital story production to maintain the momentum of EBCD. Seeking consultative support on pre‐analysed qualitative data is not uncommon [68], but we missed an opportunity for deeper engagement. Reflections from other groups suggest that formal training [69, 70], scaffolded roles [68, 71], collaborative analytic methods (e.g., framework method) [72], methodological flexibility [73] and documenting analytic decisions are important starting points for co‐analysis. We reflected that offering flexibility [68, 71] for preferred involvement levels [26, 44] in different research phases may have led to deeper co‐researcher involvement. Such phased involvement may be a consideration for future co‐production projects where resourcing and time constraints are significant issues. Reciprocating substantive research opportunities would have particularly benefitted our younger co‐researcher who felt ‘less involved’ than peers who held responsibilities in video production and project administration, respectively.

Reflecting upon other co‐production principles, we noted markers of success and areas for improvement. We incorporated diverse views into our decision‐making, but acknowledge that our expertise and connections were situated in childhood‐onset disability (e.g., cerebral palsy, Down syndrome and autism). Other principles, such as accessibility, flexibility and transparency, were largely met by our procedures. Co‐researchers raised the University's pay and email systems as barriers due to unfamiliarity and infrequent access. This was addressed through a video training tutorial and increased phone contact. Another challenge was managing the tension of age eligibility. We decided to include young people aged 8–30 years based on our grant stipulations, the need to offer developmentally appropriate materials and cater for contextual differences in paediatric and adult systems. This decision was accepted with reservations by co‐researchers, some of whom felt disappointed that research often excludes older adults with child‐onset disability. Ultimately, the team's strong relationships and collective vision for accessible cycling meant that reservations or standing aside (i.e., agreeing to compromise on a proposal) enabled decisions to flow and collaboration to occur.

## 5 Strengths and Limitations

The strengths of our paper include a clear demarcation between processes, a co‐production audit trail and illustrative materials. However, it should be noted that all of our research team were adults and no one had an intellectual or cognitive impairment. Power sharing and role reciprocity were areas for improvement in co‐production. With a larger budget, greater resourcing capacity and longer time frames, we would have supported co‐researchers to undertake more meaningful roles in qualitative data collection and analysis. This may have included scaffolded [68] learning (e.g., observe, co‐lead or lead interviews/workshops) [71], targeted training in co‐analysis and/or phased involvement in preferred research activities. Such involvement may have achieved a greater depth of co‐researcher influence.

A strength of our protocol was developing an embedded co‐design evaluation. Process evaluation has been identified as a gap in previous EBCD projects [20]. Ethical safeguards were another strength, which are shared through our supplemental file. Our protocol also had limitations. While aiming to include people with diverse disabilities, we could only include symbolic communicators (i.e., people who can share intentional messages via verbal or augmentative and assistive communication with or without a support person). This decision was influenced by ethical and methodological requirements for informed consent and rich informative accounts. This meant people with severe‐profound intellectual disability, or non‐intentional communicators, were excluded. We attempted to redress this exclusion through parent‐proxy representation. Time limited our ability to pilot or validate the young person's evaluation survey. However, survey content was influenced by a standardised outcome measure [37] and consumer evaluation with a similar target population [74]. Lastly, our accelerated EBCD may have impacted the quality of co‐designers' engagement. We detailed several strategies to retain high‐quality engagement and will explore their effectiveness through our process evaluation.

## 6 Conclusions

Our reflection may guide researchers, quality improvement leaders and consumers in exploring inclusive co‐design in disability or marginalised community groups. We found that involving co‐researchers in EBCD through a superimposed co‐production process enabled us to prepare the online setting, oversee EBCD processes and implement EBCD activities. Our experience highlighted the importance of identifying feasible deliverable(s) early, setting shared project objectives and mapping meaningful co‐researcher roles across both processes. We stretched co‐researcher involvement across a prolonged period and faced funding constraints, which sometimes led to more consultative roles. However, by sharing our process outputs, we envisage that others can advance co‐researchers' involvement in ‘doing’ EBCD activities, such as interviewing participants or facilitating workshops. Future studies should plan for early consumer involvement, offer phased involvement options and share resources to accelerate the possibility for more consumer‐led research.

## Author Contributions

**John Carey:** conceptualisation, methodology, software, validation, analysis, investigation, resources, data curation, writing – original draft, writing – review and editing, visualisation, project administration, funding acquisition. **Alicia Spittle:** conceptualisation, methodology, analysis, investigation, writing – review and editing, visualisation, supervision. **Christine Imms:** conceptualisation, methodology, analysis, investigation, resources, writing – review and editing, supervision. **Nora Shields:** conceptualisation, methodology, investigation, writing – review and editing, supervision. **Margaret Wallen:** conceptualisation, methodology, validation, analysis, investigation, resources, writing – review and editing. **Finn O'Keefe:** conceptualisation, methodology, analysis, investigation, resources, writing – review and editing, visualisation. **Miriam Joy Yates:** conceptualisation, methodology, analysis, investigation, writing – review and editing, visualisation, project administration. **Holly Skilbeck:** conceptualisation, methodology, analysis, investigation, writing – review and editing, visualisation. **Rachel Toovey:** conceptualisation, methodology, software, validation, investigation, resources, data curation, writing – review and editing, supervision, project administration, funding acquisition.

## Conflicts of Interest

The authors declare no conflicts of interest. The research team catered to the potential conflicts of interest of financial reimbursement for MISCH's co‐design services by citing their involvement in participant materials and publications.

## Supporting information

2025415 V3 supplemental file final identifiable.

## Data Availability

The data that support the findings of this study are available on request from the corresponding author. The data are not publicly available due to privacy or ethical restrictions.
