# Supplementary material for: Adapting Experience‐Based Co‐Design to Disability Research: Co‐Producing the CycLink Co‐Design Study
Source: Health Expect. 2025 Apr 28;28(3):e70276. doi: 10.1111/hex.70276 (PMC12037989; doi:10.1111/hex.70276)
Supplement: Supplementary file 1 — 2025415 V3 supplemental file final identifiable. [file HEX-28-e70276-s001.docx]

[Section A: Co-production materials 2](#_Toc183958451)

[1. GRIPP2-SF Table 3](#_Toc183958452)

[2. Co-researcher terms of reference document 6](#_Toc183958453)

[3. Sample preparation material/agenda 8](#_Toc183958454)

[4. Further examples of iterative decision-making 10](#_Toc183958455)

[Section B: CycLink Co-design materials 12](#_Toc183958456)

[1. Recruitment material 13](#_Toc183958457)

[2. Easy English participant information 14](#_Toc183958458)

[3. Plain language guide 19](#_Toc183958459)

[4. Verbal consent guide (adapted from Arscott et al (1998)^49^ 26](#_Toc183958460)

[5. Study sample frame 30](#_Toc183958461)

[6. Photo-elicitation interview materials 32](#_Toc183958462)

[7. File folder information sheet 36](#_Toc183958463)

[8. Worked Example 37](#_Toc183958464)

[9. Communication support partner briefing 39](#_Toc183958465)

[10. Advisory group preparation material (meeting 1) 40](#_Toc183958466)

[11. Content for sharing on digital display tool 41](#_Toc183958467)

# Section A: Co-production materials

# GRIPP2-SF Table

**Table S1:** Co-researchers involvement and reflections via the Guidance for Reporting Involvement of Patients and the Public- Short Form (GRIPP2-SF)^1^

| **Section and topic** | **Item** | | **Page** |
| --- | --- | --- | --- |
| Aims of consumer involvement | The aims of co-researcher involvement were threefold:   1. To develop a protocol/research materials for participants in the CycLink Co-design Study. 2. To implement the research protocol as co-researchers within a ‘project steering group’. 3. To reflect on the facilitators (+) and barriers (-) experienced during co-production. |  | |
| Methods for involving consumers | Our participatory methods were guided by six phases of co-production.^31^ We used an expression of interest form (REDCap), phone-correspondence, SMS, email, videoconferencing (Zoom), document review (OneDrive) and a terms of reference document to communicate roles and decisions.  Our reflection was developed through meeting minutes, discussion and personal reflection diaries.  Co-researchers and academic researchers and jointly reported findings in this GRIPP2-SF table. |  | |
| Results of consumer involvement | Co-researchers perceived their involvement enhanced the “person-centeredness” of the CycLink Co-design Study protocol. Co-researchers championed choice and accessibility within research materials.  Co-researchers' involvement levels ebbed and flowed throughout the study and ranged from consultation (e.g. refining qualitative findings) to partnership (digital story co-production).  Our reflections (see below) identified barriers and facilitators to co-researcher involvement. |  | |
| Discussion and conclusion | Our co-production was iterative and evolved in response to shared decisions (e.g. preferred terminology), external factors (e.g. timeframes) and consultative advice. Early and ongoing co-researcher involvement was central to the CycLink Co-design Study. |  | |
| Reflections and critical perspective | Combining co-production as the overarching research method with an EBCD process created a “complex project” that took time to “distil”. We reflected that roles and influence became more apparent in the later phases of co-production. We identified power-sharing as an improvement area for our co-production. This could be attained through a co-created terms of reference document (e.g. early pre-identification of roles, preferences and skillsets) and training on doing qualitative methods (e.g. conducting, coding or analysing interviews). This would have complemented our EBCD and offered more meaningful involvement via interaction with participants and cycling-related data.  Factors that went well (facilitators to co-production):  (+) Using existing co-production guidelines and adapting materials helped us set up our process  (+) Retaining a connection to cycling in meetings sustained our motivation/engagement  (+) Allocating significant defined roles offered a sense of contribution (e.g. video producer, administrator)  (+) EBCD’s “blueprint” of phases/stages kept us on track (i.e. offered us flexible foundations and terminology)  (+) Setting clear expectations on the scope of involvement through preparation material offered clarity  (+) Learning how to “wear different hats” through reflexivity activities supported qualitative methods  (+) Working virtually enabled flexible meeting times and communication (e.g. online document review)  (+) Providing video tutorials, phone-advice and time supported IT access (e.g. logging a timecard)  Factors that were challenging (barriers to co-production)  (-) Funding very early consumer involvement pre-grant acquisition was ethically challenging and relied on volunteerism/altruism  (-) Time to prepare for co-researcher involvement (e.g. grant applications, making training/project materials)  (-) Developing co-researcher skills and confidence to lead research activities in over a short period  (-) Tight turnaround times and budget limitations affected role allocation and stress levels (e.g. deadlines)  (-) Casual employment over an extended time affected familiarity with study progress and university IT  (-) Delegating qualitative roles whilst developing PhD candidate’s learning and ensuring rigor/quality  (-) “Juggling the chaos” of competing priorities, changing roles and offering “the lived experience perspective” was sometimes emotionally stressful  (-) Stipulations of prospective grant (i.e. target population age <30 years) led to compromise for inclusion criteria  Recommendations for future co-production:   - Flexibility for synchronous (e.g. meetings) and asynchronous (phone calls, SMS) communication - Early agreement on project scope and opportunities for phased involvement, such as choosing deeper involvement for preferred research activities and allocating resourcing to develop capacity - Keeping team members updated on overall project updates through regular communication (e.g. sharing the role of monitoring the study’s inbox, distributing progress summaries) - Sharing resources, reflections, toolkits and guidance on using qualitative methods with co-researchers (e.g. roles, methods and rigour in co-analysis) |  | |

Footer: Please see main text for full citation ^1)^ Staniszewska et al 2017; ^31)^ Strnadová et al 2020

# Co-researcher terms of reference document

| **Co-design phase & timepoint** | **Proposed Co-Researcher Activities** | **Example opportunities: where your voice can land** |
| --- | --- | --- |
| **Planning**  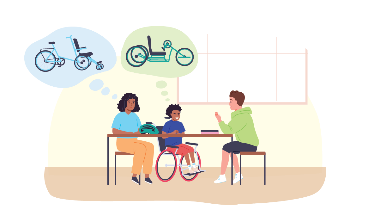  **Quarter 4**  **2021- Quarter 1 2022** | - Complete ethics research training (2 hours). - Complete basic training in Point of Care Foundation Experience-Based Co-Design Toolkit (Optional) - Reflect: your interests in the project, best use of your expertise/skills/time. - Keep a reflexive log (expectations, hopes, interests, learning points) - Help review the ethics application (2-3 hours). - Explore ways to gather experiences (e.g. journey mapping, photo elicitation, video analysis)   **Goals:**   - Complete human research ethics training - Review university ethics application | - Help choose research question, methods, tools/outcome measures used - Collaborate on experience-gathering interviews (e.g. choosing methods, making an interview guide) - Help make a ‘sample frame’ (criteria for representation and people who can get involved in the study) - Collaborate on participant-facing information/material for the study (e.g. posters, social media advertisement, information sheets) |
| **Experience Gathering**  **(Interviews)**  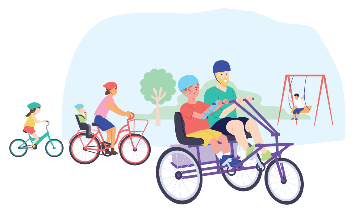  **Quarter 3 2022** | - Help make sense of people’s experiences and develop ‘touchpoints’ (key areas for improvement in a person’s experience) - Learn about qualitative research methods   **Goal:**   - Help interpret cycling stories (experiences, key themes, barriers, facilitators) that we can share in the co-design workshops | - Help decide on the selection of participants for interviews and co-design workshops - Help conduct a virtual observation or experience-gathering activity (e.g. interview) - Help edit/create a ‘digital story’ or visual experience/journey map on participating in cycling |
| **Co-design**  **(Workshops)**  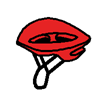  **Quarter 4 2022** | - Help present the cycling story to the co-design teams - Help decide on objectives for the co-design teams   **Goal:**   - Help interpret the outcomes of the co-design process and how we can present the CycLink resource/program/pathway | - (To be confirmed) Help analyse the direction of decisions in workshops (e.g. where the decision originated and ended) - Learn more about designing ‘complex interventions’ and how CycLink is designed - Present to others: explain the cycling story we formed to co-design team |
| **Celebration Event**  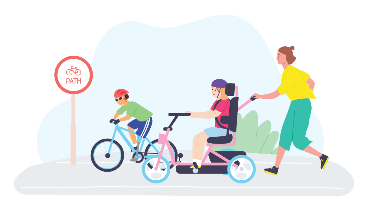  **Quarter 1 2023** | - Help decide on how CycLink is presented to others (e.g. manual, resources) - Collaborate on how we explain our findings to others   **Goals:**   - Read and review articles that the team produce - Review visuals (infographics) that the team produce. | - Help interpret the study’s results (e.g. evaluation of the process and experiences) - Help present to others (e.g. at a community of practice or conference) on your involvement and the study findings |

# Sample preparation material/agenda

**Preparation Material**

**Online meeting:** 13/12/2021 chat by Zoom 1-2pm


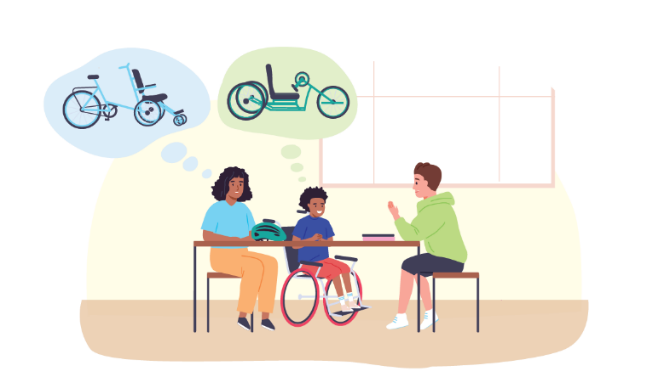


**Our key words are:**

Participation (all) Early opportunities (MY) Journey (HS)

Empowerment (FO’K) Rehab to Recreation Link (JC/RT) Collaborate (all)

**Our BIG goals at this phase:**

- Complete research ethics training
- Decide on experience-gathering methods
- Review ethics application

**Focus for the meeting:**

Today we’ll talk about ways to gather peoples’ experiences of learning to cycle.

We’ll discuss **WHO** (co-design participants), **HOW** (qualitative methods) and **WHAT THEN** (output/deliverable from co-design) we can do when we gather peoples’ cycling stories.

**Ways that you can prepare for the meeting:**

1. What questions would you like to ask young people with disability, family members or cycling providers?

**Write down your questions.**

1. Who do you feel should be represented and included on the sample frame or “wish-list”?

**Write down characteristics important for diversity in the sample frame.**

1. Keep working on your biography for ethics and send us a portrait photo.

**Please send them by 5pm 23^rd^ December 2021.**

1. **WHO (additional Word Document was attached)**


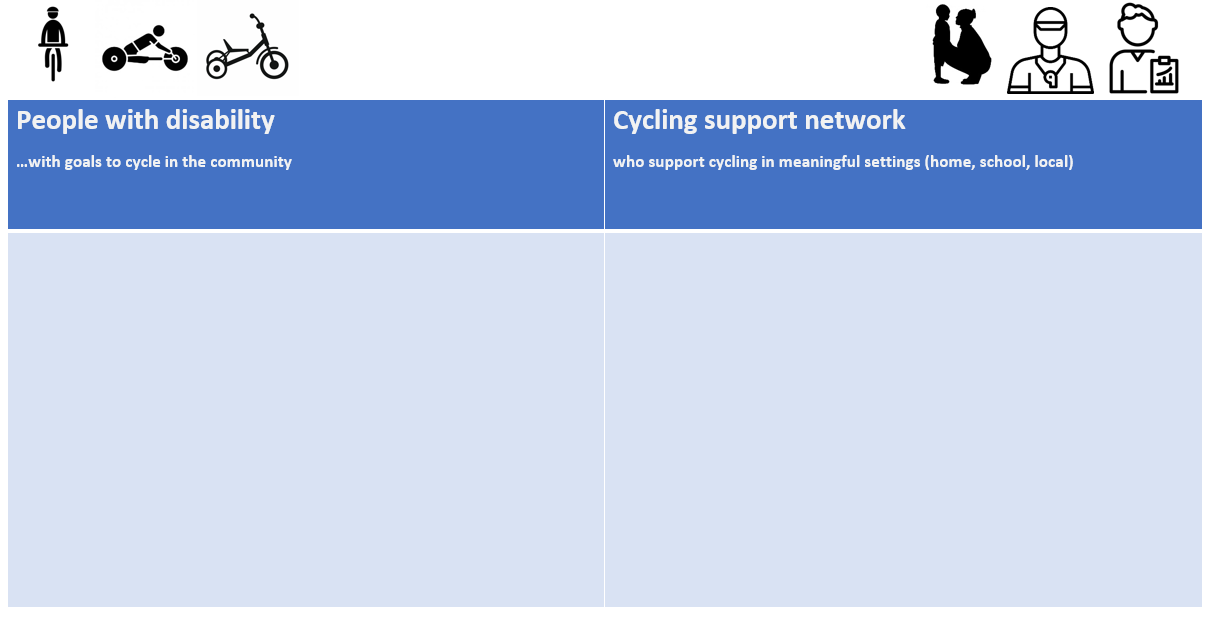


1. **HOW**

Our co-design convenors are helping us choose ways to gather peoples’ experiences. They have suggested using semi-structured interviews* which include photographs to generate discussion during a Zoom call.

*this means a flexible interview guide of possible questions.

***Is this acceptable to you?***

***What questions would you ask?***

**Some Examples questions from a semi-structured interview:**

*Areas of interests: starting out, goal, challenges and enablers, connection.

- How did you get involved in cycling?
- Why was cycling important to you?

Prompts: what was your goal, who did you plan to cycle with, when would you plan to cycle?

- What were the challenges in getting started with cycling?
- What helped you to reach your goal?

# Further examples of iterative decision-making

**Figure S1:** Developing our study’s sample frame.


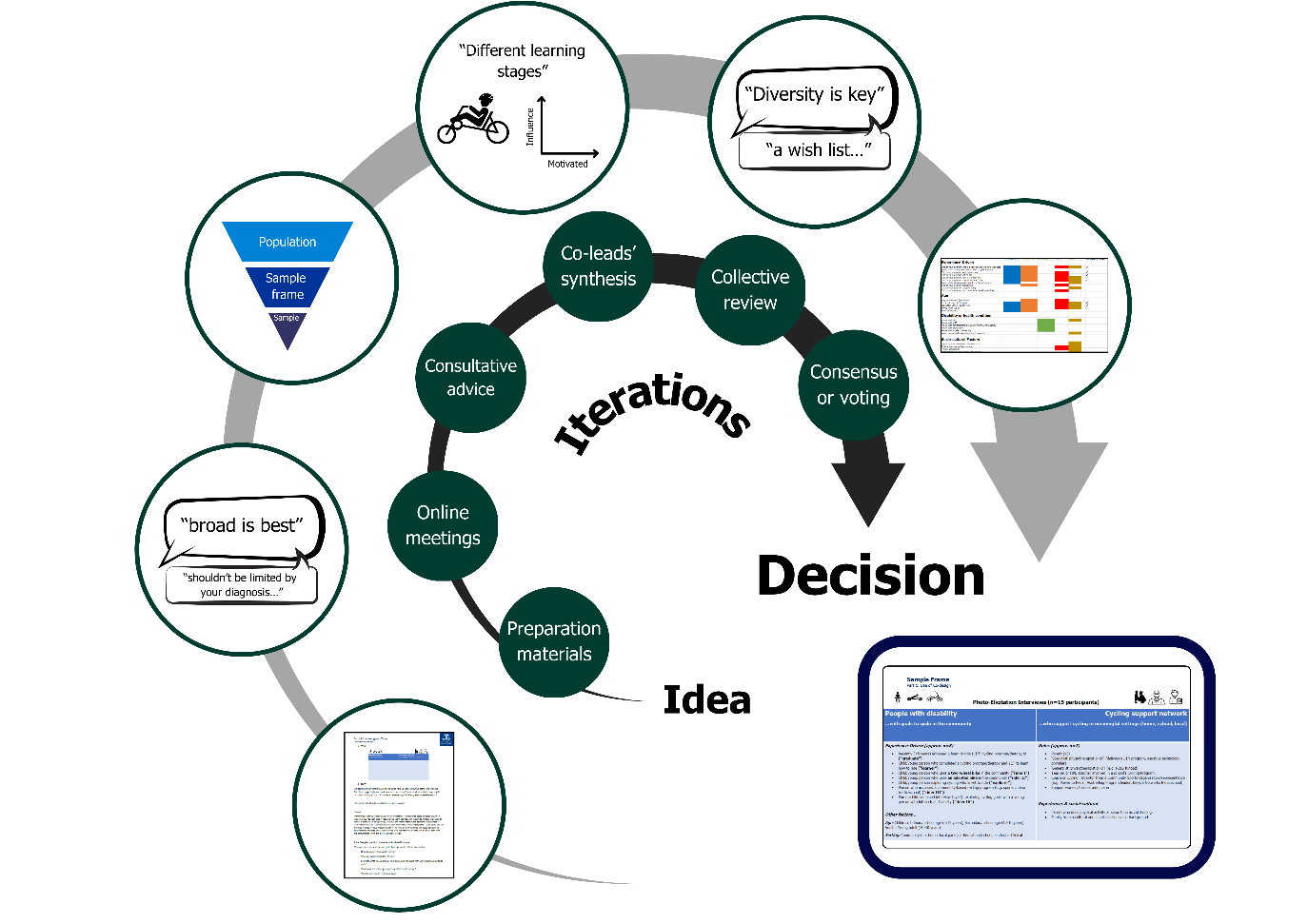


Notes: The inner dark arrow represents our decision-making process. The outer light arrow illustrates examples of an agenda (box 1), co-researchers’ discussion points (box 2), senior researcher’s explanation of sample frames (box 3), lead researcher’s investigation of learning levels (e.g. Australian Physical Literacy Framework) and EBCD’s Influence-to-Motivation tool (box 4), reaching consensus on population and use of a sample frame (box 5), voting results charted in Excel (box 6) and the final sample frame for experience-gathering phase (box 7).

**Figure S2:** Deciding on our study’s evaluation survey tools.


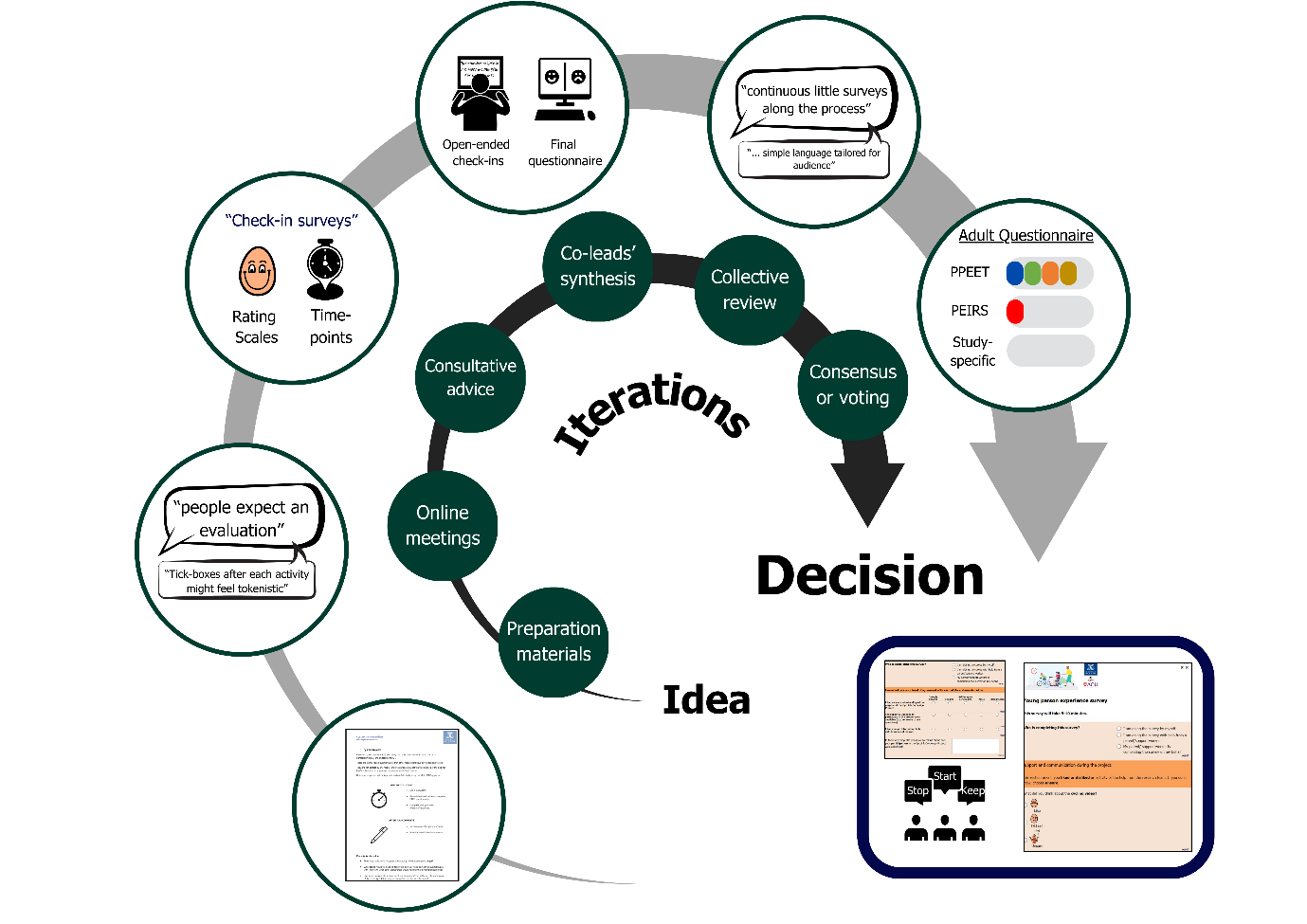


Notes: The inner dark arrow represents our decision-making process. The outer light arrow illustrates examples of an agenda (box 1), co-researchers’ discussion points (box 2), consultative advice from evaluation team and speech pathologists (box 3), lead researcher’s synthesis of including both open-ended surveys and standardised questionnaire (box 4), co-researcher comments after pilot-testing surveys (box 5), voting for the adult evaluation questionnaire (box 6) and the final evaluation questionnaires and check-in surveys (box 7).

*Picture Communication Symbols^®^ (PCS) is a trademark of Tobii Dynavox LLC.*

*All rights reserved. Used with permission.*

# Section B: CycLink Co-design materials

# Recruitment material

Social media advertisement


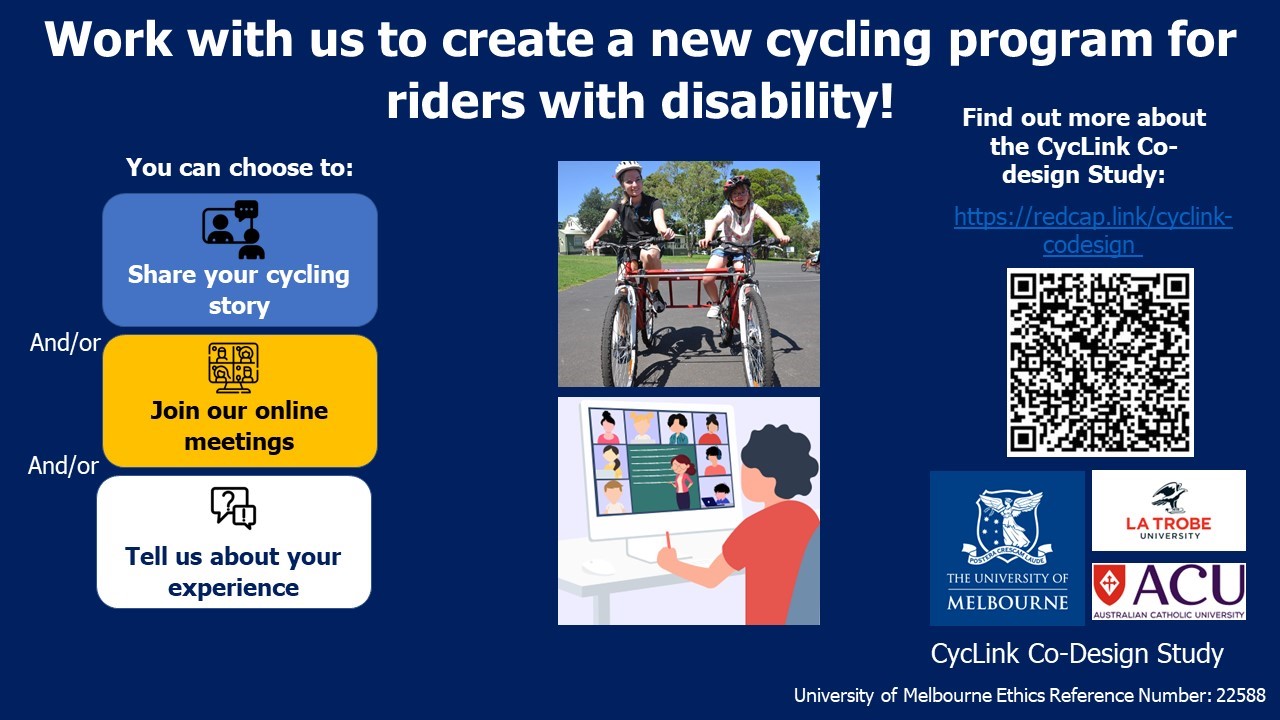


Poster advertisement


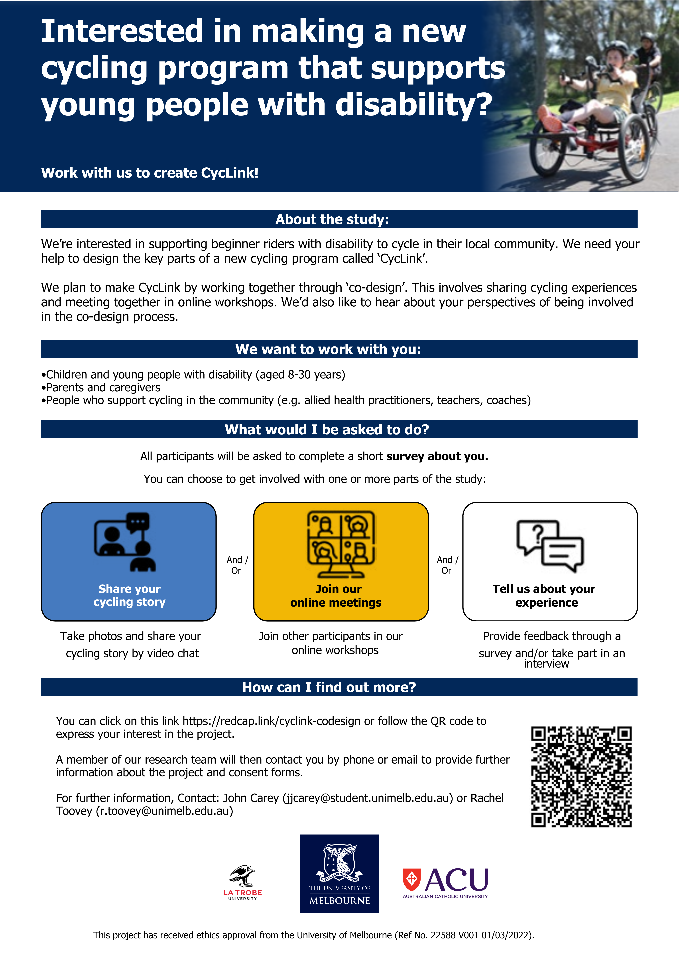


# Easy English participant information


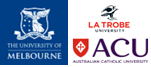


**Information about the research project**

Easy Read Information

**Are you interested in cycling?**

**We would like you to join our research project.**

Our team includes researchers, people with disability, a parent and therapists. Some of us are pictured here but you will also meet other members of our team.

| 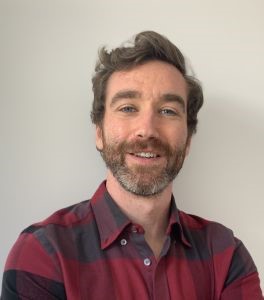 | 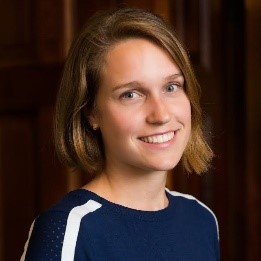 | 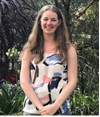 | 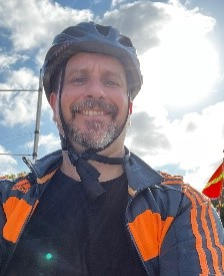 | 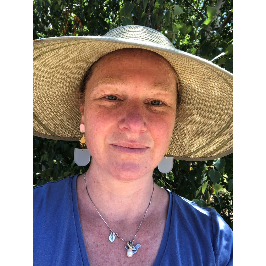 |
| --- | --- | --- | --- | --- |
| John | Rachel | Holly | Finn | Miriam |

**About our research**

| 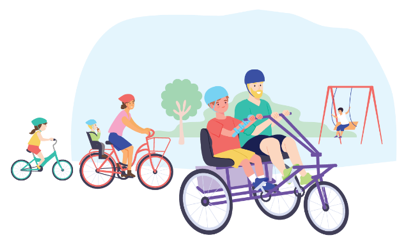 | Riding a bike can be fun and is good exercise!  You can cycle with friends or family.  There are lots of different types of bikes; like trikes or handcycles.  But there are not many cycling programs for young people with disability. |
| --- | --- |
| 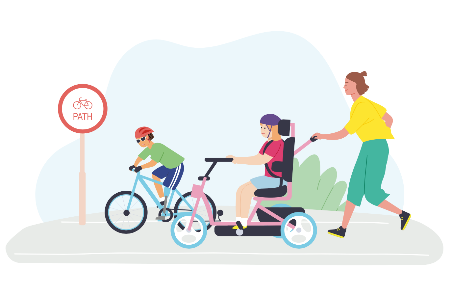 | **We want your help to make a new cycling program.**  **We think this might help more young people to enjoy cycling in the community.** |
| **Who can take part?**  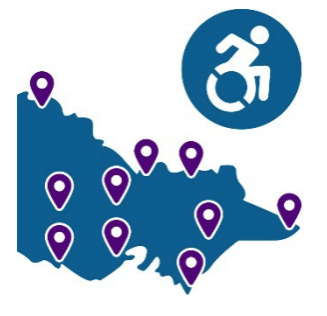 | We are asking for your help because:   - You are a young person with disability - You live in Victoria - You are aged between 8 and 30 years old |
| 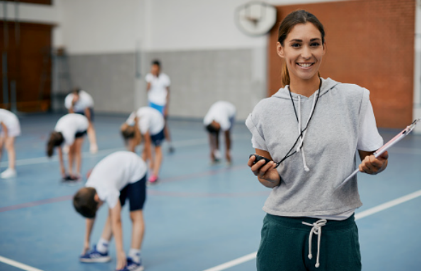  We will also ask parents and carers, therapists, coaches and teachers in Victoria to take part in a different group. | |

**How can I get involved?**

There are 3 different ways to take part.

You can choose to do 1 option or more.

| 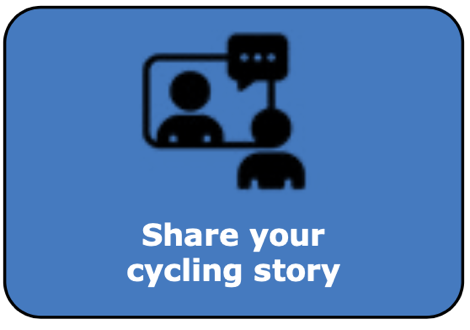 | **Option 1: Photo-interview**   - We will ask you to take photos about your bike-riding. - We will give you information about taking photos safely and responsibly. - We will ask you about your photos in a video chat which lasts about 30 minutes. |
| --- | --- |
| **AND / OR** | |
| 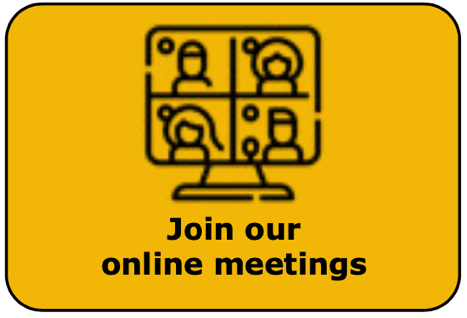 | **Option 2: Online meeting with other young people**   - We will do online meetings to talk about different ideas for making a new bike-riding program.      - The meetings will be with us and other young people in the project who are a similar age to you. - You can take part in 1 or 2 group online meetings. - We will send a worksheet to prepare for the meeting. Each meeting takes 30-45 minutes. |
| **AND / OR** | |
| 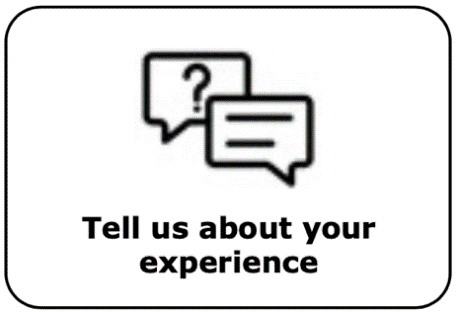 | **Option 3: Survey and/or video chat about being involved in our project**   - Do an online survey and/or video chat with a researcher about helping to make the cycling program. - The survey and researcher will ask you what it was like to share your cycling story or discuss ideas in the group. - The survey will take 5-10 minutes. The video chat will last about 30 minutes. |

| 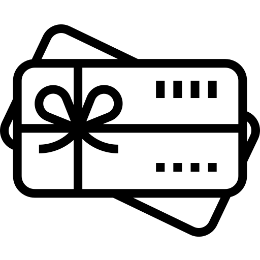 | To thank you for your time, we will give you a $25 gift voucher for every interview you do or every meeting you go to. |
| --- | --- |
| 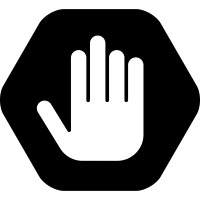 | Taking part is up to you. You can choose to stop at any time.  You can tell us if you want to leave part of a chat or meeting. You can tell us if you want to stop doing the research all together. |
| 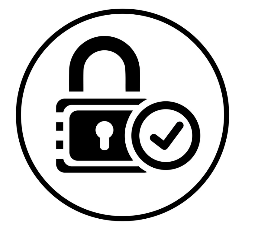 | We won’t share your real name with anyone. Information about you will be kept private. You can choose whether you’d like to give us your permission to share your photos. |
| 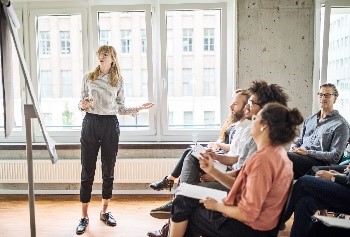 | At the end of this study, we will talk about the project with other people with disability, researchers and professionals but people won’t know you took part. |
| 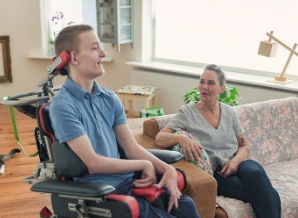 | If you are interested, please let your parent or guardian know. We will then talk to you and your parent or guardian about how you can get involved. |
| 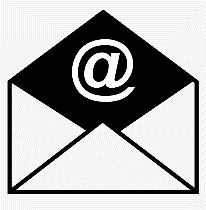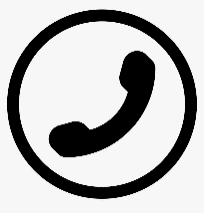 | **Please contact us with any questions:**  **Rachel’s** contact: [rtoovey@unimelb.edu.au](mailto:rtoovey@unimelb.edu.au)  **John’s** contact: [jjcarey@student.unimelb.edu.au](mailto:jjcarey@student.unimelb.edu.au) |

# Plain language guide


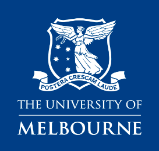

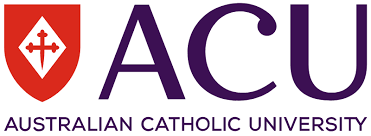

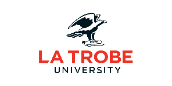


**Plain language statement**

Information for parents and carers whose child is interested in participating

The University of Melbourne- Department of Physiotherapy
School of Health Sciences, Faculty of Medicine, Dentistry and Health Sciences.


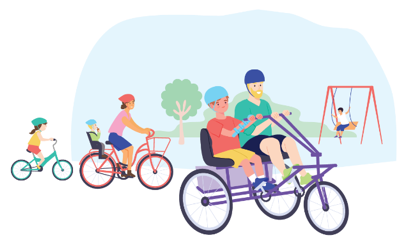


**The CycLink Co-design Study:**

**Co-design as a route towards cycling participation in young people with disability**

**Responsible Researchers:**

Dr Rachel Toovey: [r.toovey@unimelb.edu.au](mailto:r.toovey@unimelb.edu.au) / PHONE

John Carey (PhD student): [jjcarey@student.unimelb.edu.au](mailto:jjcarey@student.unimelb.edu.au) / PHONE

**Introduction**

Thank you for supporting your child’s interest in this research project. The following few pages will provide you with further information about the project, so that you can decide if you would like your child to take part in this research.

Please take the time to read this information carefully. You may ask questions about anything you don’t understand or want to know more about. Your child’s participation is voluntary. If your child doesn’t wish to take part, they don’t have to. If your child begins participating, they can also stop at any time. As a parent or caregiver, your role may extend to supporting your child’s participation in the study. This can include:

- Helping them to complete **online tasks** such as using Zoom, sending/receiving emails and uploading/downloading files related to the study.
- Supporting them in interviews as a **familiar communication partner** and assisting with interview **engagement**.
- During interviews/workshops, we will direct our questions towards your child to hear their response. Where appropriate you can add your own interpretation or perspective during these conversations. We will record and use your comments to help support their voice, unless you tell us not to.

**What is this study about?**

Cycling is a popular way to get active. Cycling has health and social benefits, as well as environmental benefits as a form of active transport. Participating in cycling is available for some, but not all young people with disability. We know that access to a suitable bike and cycle skills programs are good starting points for cycling participation. However, few cycling programs have focused on ways to integrate cycling into family, home and community life.

We want to work with young people with disability, their families and practitioners to create the ‘guiding principles’ of a new cycling program. By guiding principles, we mean the key features of the program. These principles may offer strategies to address possible problems and identify solutions when supporting young people with disability to cycle in their local community.

We believe working together will provide the best outcomes for young people with disability. Experience based co-design is a way to work together. We want to understand how we work together using experience-based co-design to develop this intervention. We also want to find out what being involved in co-design is like for young people with disability, their parents and carers, and practitioners. Understanding how we work together through co-design might help other co-design projects to be accessible and engaging.

**Why is my child being invited to take part?**

We are inviting young people with disability (aged 8 – 30, any type of disability) who live in Victoria and/or their parents and carers to participate in this research.

We need young people with disability to take part in this study as they know what works well for them. Their contribution will mean that the program has a better chance of meaningful outcomes for all involved. It will help us to break down barriers to inclusion.

We are inviting other community members who support young people with disability to cycle to participate. This includes parents, allied health professionals, support workers, educators, cycling coaches and sport and recreation professionals. You can choose to participate separately and represent your perspectives as a parent by accessing the study’s expression of interest.

**What will my child be asked to do?**

We will first collect basic information about your child via a 10-15 minute survey. We collect this information so that we know about who participated in the study, and to make participating accessible for your child where possible. Then, your child can be involved the study in one or more of the following three ways, depending on their age.

| 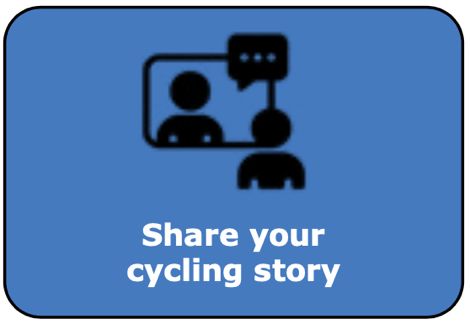  **AND / OR** | **‘Photo-interview’: Video interview involving photo methods**  Children and young people (aged 8-30 years) can take part in a video interview with a researcher to share their experiences of cycling.  To prepare for the interview, we will ask your child to take cycling-related photographs on a smartphone or tablet*. We will provide a tip-sheet and discuss how to take photos safely and responsibly. We will ask for 3 photos to be selected and shared with us for an online interview.  The photos will help guide the interview. With your additional consent, these photos may be included in a video to share participants’ stories.  We anticipate gathering photos will take 1-3 hours while the interview will take approximately 30-45 minutes. Your child can attend the interview on their own, or with your (or another persons’) support if needed. The aim of the interview is to hear the voice of the children and youth.  *If your child cannot access a camera on a mobile device or access may cause behavioural challenges; tell us. We can provide a mounting stand, adapted switch to aid access or a polaroid camera and film for taking photos. |
| --- | --- |
| 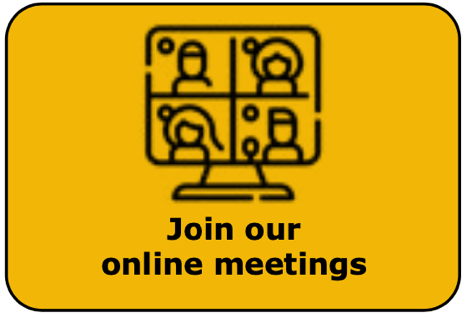  **AND / OR** | **Online group workshops**  Children aged 8-15 years can also take part in one or two online co-design workshops. These workshops will involve 5-10 other children and young people of a similar age.  The purpose of these workshops is for children and young people to have a say in what the cycling program looks like in fun and accessible way.  Your child can attend these workshops on their own or with a parent or support-person present. We will provide preparation material to your child by post or email about 10 days before the workshop.  Each workshop will take 30 – 45 minutes. The workshops will be led by members of the research team who are experienced with working with young people. These workshops will take place outside of school hours over an 2–3-week period in [**insert relevant months / year**]**.** |
| 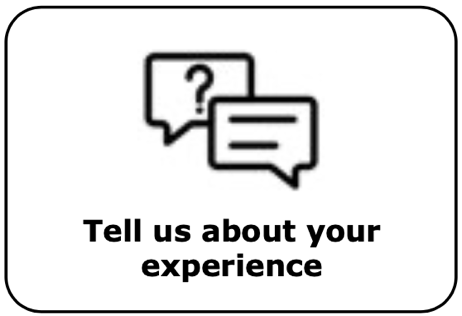 | **Online survey and/or video interview about co-design experiences**  Your child can tell us about their co-design experiences through an online survey and/or a video interview.  The once-off survey will take approximately 5-10 minutes and will be sent to you to following your child’s final involvement in the study.  The interview will take approximately 30 minutes. It will occur within four months of the online meetings. This interview will be with a researcher who was not involved in the other parts of this study, and who is experienced with working with children and young people.  Your child can attend the interview or do the survey on their own, or with your (or another persons’) support if needed. The aim of both the interview and the survey is to hear the voice of young people. |

All study activities will be virtual, done either via online surveys or videoconferencing using Zoom. Participants under 18 years old will need to access Zoom via the account of their parent or carer as Zoom only allows accounts for people over 18 years.

A $25 voucher will be given to each participant for each occasion they are involved as a gesture of appreciation. For example, if your child shares their cycling story, attends two online meetings and shares their co-design experiences, they will be given a $100 voucher (4 occasions).

**What are the possible benefits?**

We are doing this study for research purposes. Our aim is to work with young people with disability, their parents/caregivers and relevant community members to come up with guiding principles that will form the foundation for a cycling program. This means that the study will **not** directly benefit you or your child.

However, our intention is that the guiding principles that emerge from this project will inform a cycling program which we will run and evaluate in the future. The aim of this program will be to support young people in Victoria to participate in cycling, and this may be of benefit to your child and your family in the future.

**What are the possible risks?**

We think the risks for participating in this study are low. We will discuss ideas and issues about disability or challenges for cycling participation. We will acknowledge the strengths and possibilities of young people throughout the discussion. Interviews and meetings will be with researchers with significant clinical experience of working in paediatric disability. There is a small risk that questions may be upsetting for some participants, or that sensitive topics are raised and shared with the research team. All participants will be provided with general information regarding support. If your child does become upset, your child will be given the option to stop the interview. If appropriate, the research team will encourage you to follow this up with your child’s appropriate health practitioner.

**Does my child have to take part?**

No. Participation is voluntary. You can withdraw your child at any time. If your child leaves the study, we will use any information already collected unless you tell us not to. In rare cases where the research team suspects forced involvement (i.e. coercion from a caregiver) or signs of disengagement/distress we will organise a debrief call. During this call we may discuss options for ongoing participation or potential withdrawal from the study.

**Will I hear about the results of this project?**

Following the co-design workshops, we will invite you and/or your child to a celebration event to inform all participants about the key themes gathered from the interviews and the co-design workshops. We will also send you and/or your child a summary of the results via email at the end of the project.

**What will happen to information about my child?**

We will collect and use information about your child for research purposes. The storage of your child’s information will depend on how they participate in the study:

**Consent and Surveys**

Consent and survey data will be securely collected and stored via The University of Melbourne’s password protected REDCap database. Some young people may provide verbal consent and in these cases, the audio recording of consent will be retained. This data will be transferred to password protected files in the University of Melbourne’s secure server and will only be accessible to limited researchers on the investigation team.

**Interviews**

The photo-interview (option 1) and co-design experience interview (option 3) will initially be stored securely on the research team’s University of Melbourne Zoom accounts. We will use the Zoom platform to generate an audio transcription. This means a computer will be used to generate a full **written copy of the interview**. Following this process, the recordings and transcriptions will be downloaded to the University of Melbourne server and deleted from the Zoom accounts. We will use the audio recording to check the written transcript is accurate. We will remove identifying details such as your child’s name, date of birth, email and address and give your child a code name during this process. This code name will also be used for analysis and reporting. Once the research team have checked and analysed all of the interviews, all audio and video recordings will be deleted from the University of Melbourne server. Only the typed up transcripts and audio recording will be kept. If your child is invited to do a photo-interview, we will also ask your permissions for use of their photo(s) outside of the research team for educational and research purposes.

**Online Workshop Recordings**

We will collect and analyse the video recordings from the online workshops (option 2). We will use the video recording to check for accuracy of content, contributions and to type up minutes. We may gather notes or conversation snippets to highlight key parts of the co-design process. We will remove identifying details such as your child’s name, date of birth, email and address and give your child a code name during this process. You can tell the workshop facilitators if you would like to have your child’s first name included in project materials where their idea or contribution is shared with others. Otherwise we will use the code name.

**Privacy and long term storage**

Except for where you have provided additional consent for your child’s photo/s to be shared, the only people who may access your child’s **identifiable** information are:

The University of Melbourne and Australian Catholic University researchers who are involved in data collection (consent, interviews, meetings)

The University of Melbourne Human Research Ethics Committee

To advance science and health, we may also share your child’s **de-identified** data with others for ethically approved research. We have put security measures in place to protect your child’s data if and when we give it to other people. All sharing of data will be done using the University of Melbourne’s secure server.

We are required to keep information collected as part of a research project for 15 years after the youngest participant turns 18. The research information may be destroyed or kept indefinitely after this time. You have the right to access and correct the information we collect and store about your child. This is in line with relevant Australian and/or Victorian privacy laws. Please contact us if you would like to access this information.

**Funding for this study**

Funding for this study is through a Melbourne Disability Institute Seeding Grant. This funding will in part be used to pay for Dr Jennifer Bibb and Prof Victoria Palmer to facilitate the co-design workshops. These members of the research team will not be involved in the collection or analysis of data related to your co-design experiences.

**Discussing this study with your child**

We recommend discussing this project with your child to find out whether they would like to participate. If you think that your child is mature and able to understand this form, we ask that they read this document, and indicate consent as a mature minor. Otherwise, we encourage you to discuss the project with your child using the Easy English statement.

**Where can I get further information?**

If you would like more information about the project, please contact the responsible researchers; John Carey: [jjcarey@student.unimelb.edu.au](mailto:jjcarey@student.unimelb.edu.au) /(phone) or Dr Rachel Toovey: [r.toovey@unimelb.edu.au](mailto:r.toovey@unimelb.edu.au) /(phone).

**Who can I contact if I have any concerns about the project?**

This project has human research ethics approval from The University of Melbourne (Ethics Reference Number: 22588). If you have any concerns or complaints about the conduct of this research project, which you do not wish to discuss with the research team, you should contact the **Research Integrity Administrator**;

Office of Research Ethics and Integrity, University of Melbourne, VIC 3010.

Tel: +phone or Email: [research-integrity@unimelb.edu.au](mailto:research-integrity@unimelb.edu.au).

All complaints will be treated confidentially. In any correspondence please provide the name of the research team and/or the name or ethics ID number of the research project.

# Verbal consent guide (adapted from Arscott et al (1998)^49^

**Verbal consent script for potential participants with cognitive impairment or intellectual disability**

1. **Introduction**

Thanks for coming along. Today we will talk about a cycling project that you can **choose** to do. Please feel free to ask **questions** throughout this chat. You can take a **break** if you need; just tell us.

**First,** I’d like to tell you about the project. We will look at the project information together.

**Then,** we can decide if you’d like to take part.

*Researcher shares the Easy Read Information via Zoom’s share screen feature.*

*During pre-information reiterate that: a) researchers will contact by study-specific email and b) data storage and privacy information.*

1. **Confirmation of preferred communication method for yes/no response**

Before we work out if you can take part in the study, we need to go through a couple of things. These may seem tedious and unnecessary; however, they are requirements of the university.

First, we need to work out a reliable Yes/No response that you can do without assistance.

How do you communicate “Yes” and “No”?

*Await response.*

*Support person may confirm best way to ascertain Yes/No response.*

Ok, now we have established how you can communicate Yes and No, I have a few questions for you.

1. **Participant details**

*Researcher to check if potential participant has completed REDCap expression of interest form and verify eligibility and identification.*

1. **Ability to give consent**

Now I need to ask, are you happy for me to video record this?

*Researcher to video-record consent process.*

As you are with _____ (name of parent, guardian or carer) today, I want to stress that I am really interested in what *you* want to say. I will only ask ____ (name of parent, guardian or carer) to revoice when they know what you are saying. You can ask them to explain something further, but when this happens, I will check with you to confirm what they have said matches what you intended.

*Assess ability to give consent*

I am going to ask you five questions that will require Yes or No answers. These next five questions are to make sure you have looked over the project information, have understood it, and want to take part in this project. Please answer them to the best of your ability with a simple Yes or No.

1. Do you have to do this study? (**No**- Participation is voluntary)
2. Can you stop doing this study at any time? (**Yes**- You can stop at any time, you just need to say so. You can skip parts if you are not comfortable)
3. Will we send you information about the study by email? (**Yes-** we will use email to tell you about different parts of the study. The four different parts of the study include: 1) a photo-interview; 2) online workshops; 3) short feedback surveys; and 4) feedback interview)
4. Will we use a pretend name for you when we write up the findings? (**Yes**- we will use a pretend name **outside** of the study when we write up a report or tell others about the study at a conference)
5. Will information about you be kept private? (**Yes**- we will store the information in password-protected computers on the University of Melbourne internet)

*If all questions are answered correctly:*

Great, from the answers you have just given I can tell that you have read the Information Sheet and can consent to being involved.

Do you have any questions about the project?

OR

*If any questions are answered incorrectly:*

It seems that you have trouble with understanding the information sheet. Would you like to read the information sheet again? I can go through it with you and try these questions again.

OR

*Try 5 questions above again – if still incorrect:*

We know that it’s difficult to do this part. If you would still like to do this study, we can speak parent or guardian about how your family could take part (e.g. expert by experience).

1. **Consent**

*Researcher to share screen with participant and caregiver.*

**Do you give consent to participate in this project in the following ways?**

| 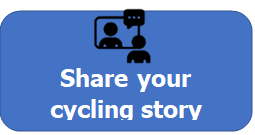 | ​​☐​‘Photo-interview’  (Video chat with photos) |
| --- | --- |
| 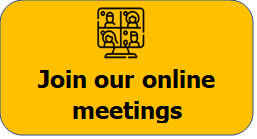 | ​​☐​Online group meetings with young people and researchers |
| 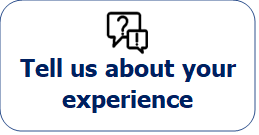 | ​​☐​Video interview about my co-design experiences    ​​☐​Survey about my co-design experiences |

**Do you give consent to for any of these other options?**

| 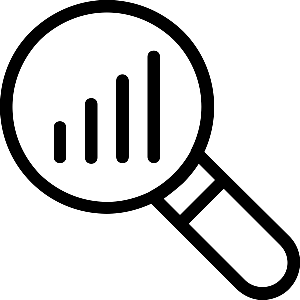 | ​​☐​ I consent for my information collected in this study to be used in related ethically approved research in future | | |
| --- | --- | --- | --- |
| **Do you give consent for any of your photos that you provide in this study to be used in the following ways:** | | | |
| 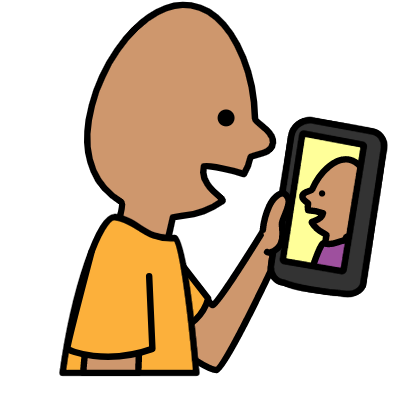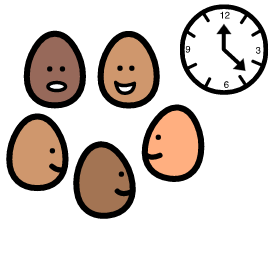 | | | ​​☐​To be shared with others in this research project |
| 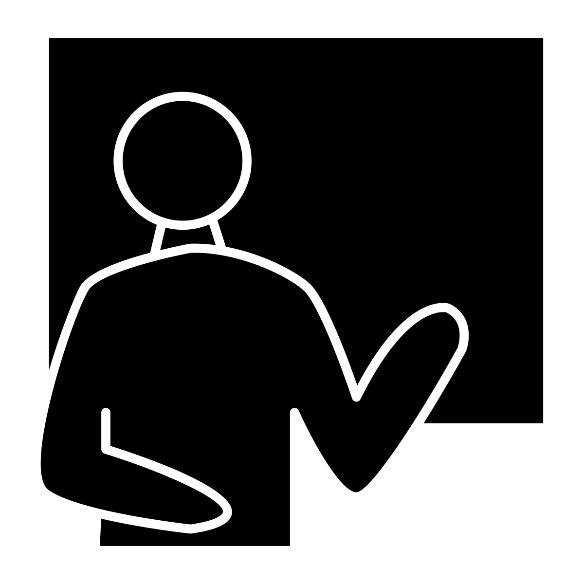 | | ​​☐​For educational and publication purposes associated with this project (e.g. teaching, conferences and supplementary resources to journal articles) | |
| 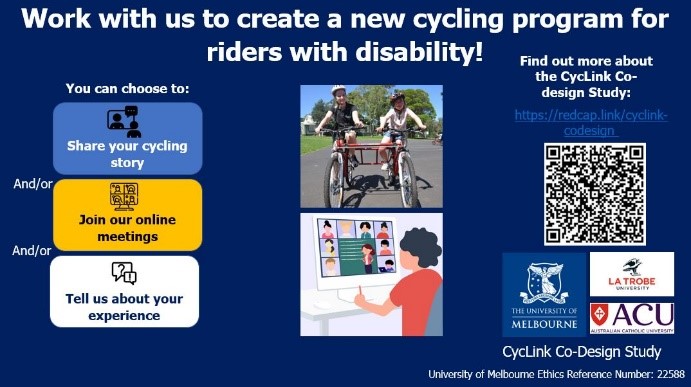 | | ​​☐​Promotional materials (e.g. information brochures about the cycling program) | |
| 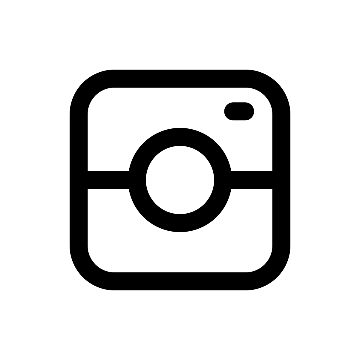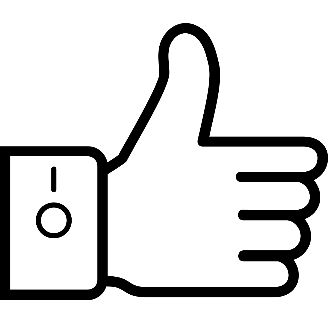 | | ​​☐​On social media (e.g. on Twitter, Facebook and LinkedIn) | |

*Picture Communication Symbols^®^ (PCS) is a trademark of Tobii Dynavox LLC.*

*All rights reserved. Used with permission.*

# Study sample frame


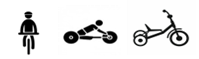

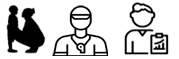
**Photo-Elicitation Interviews (n=15 participants)**

| **People with disability**  **…with goals to cycle in the community** | **Cycling support network**  **…who support cycling in meaningful settings (home, school, local)** |
| --- | --- |
| ***Experience-Driven (approx. n=8)***   - Recently (<5 years) accessed a learn to ride (LTR) cycling program/therapy to **(“graduate”)** - Child/young person who completed a cycling program/therapy and YET to learn how to ride **(“learner”)** - Child/young person who uses **a two-wheel bike** in the community **(“rider I”)** - Child/young person who uses **an adapted bike** in the community **(“rider II”)** - Child/young person exploring cycling who is yet to ride **(“explorer”)** - Person who accessed a community-based cycling program (e.g. sports club or traffic-school) **(“rider III”)** - Parent-child combined interview (dyad): exploring cycling goals with a young person with intellectual disability **(“rider IV”)**     ***Other factors…***  ***Age:*** Children: Primary school age (5-12 years), Secondary school age (12-19 years). Adults: Young adult (19-30 years)  ***Setting:*** Community (i.e. home, local parks) > Education (school, kinder) > Clinical | ***Roles (approx. n=7)***   - Parent (x2) - ‘Specialist’ physiotherapist or OT (delivers a LTR program, assistive technology provider) - ‘Generalist’ physiotherapist or OT (e.g. NDIS funded) - Teacher or HPE Teacher involved in a school’s Cycling Program. - Coach or cycling instructor from a Community Sports Organisation Representative (e.g. Power to Pedal, AusCycling Program leader, Bicycle Networks Ride2School) - Support worker, teacher aide, carer     ***Experiences & social-cultural***   - Parent who does physical activity at same time as child riding. - Family from a cultural and linguistically diverse background   HPE= Health and Physical Education  LTR= Learn To Ride  NDIS= National Disability Insurance Scheme  OT= Occupational Therapist |


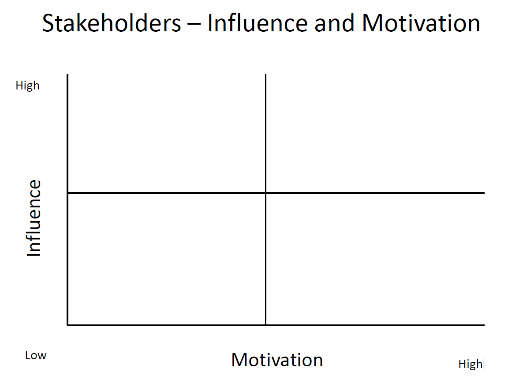
**Co-design Meetings (*n=* approx. 25-40)**

- Adult co-design groups (Lived experience and Community Partners): *n*=8-16 adult participants
- Children’s advisory panel (with Rachel and John): *n=*5-10 participants (aged 8-15 years)

**Image reference:** EBCD ‘Stakeholder Mapping’ Grid

| **People with disability**  **…with goals to cycle in the community** | **Cycling support network**  **…who support cycling in meaningful settings (home, school, local)** |
| --- | --- |
| **Lived Experience Group**  **At least 4+ people with lived experience of disability:**   - >2 people aged >18 years with disability who can take part in group discussion - >2 parents of children with disability (including cognitive impairment or intellectual disability)   *Preferences:*   - Greater representation from people with lived experience, or at least equal numbers to community partners.     **Advisory Panel**  At least 5 participants aged 8-15 years with different cycling experiences. | **Cycling providers**  **At least 4 cycling providers/community partners:**   - OT - Teacher - Physiotherapist - Cycling advocacy oragnisation (e.g. Bicycle Networks, AusCycling VIC)   *Preferences:*   - Experience in teaching traffic skills or implementing community mobility plans - High motivation to be involved and high-medium influence for driving change. |

# Photo-elicitation interview materials


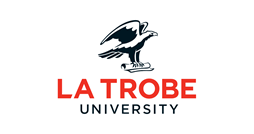

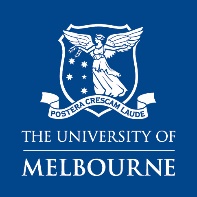

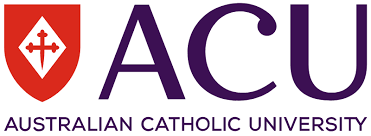


**Photo-Interview Tip-sheet**

Department of Physiotherapy
School of Health Sciences, Faculty of Medicine, Dentistry and Health Sciences.

**Photo-interviews for children & young people**

Thank you for taking part in a photo-interview.

To get ready for our ‘Zoom’ interview we’ve asked you to take **three digital photos** using a mobile device (e.g. phone or iPad). We also want you to tell us about why you took the photo in a **short sentence**.

You may need to ask a family member for permission to use their device.

They can help you send the photo and sentence by email.

You can choose if you’d like your photo(s) to be used in other parts of the study. For example, shared in a meeting or in a video (digital story).

**What should the photos be about?**

This part of the study is about your experience starting out in cycling.

*Your photos should tell us a little about* ***YOU****, how cycling makes you* ***feel****, and* ***where*** *you like to ride (now or in the future).*

Your photos might include:

- Important people, places, or achievements on your cycling journey
- Places you cycle in your everyday life, at weekends or during school holidays
- What makes cycling easy to do, or even a challenge, when you ride near home, in local parks or on footpaths

**What else do I need to consider?**

Your photos can include other people- like your family or friends.

You just need to make sure that anyone in the photos gives us their permission.

They can do this by filling out a **photo consent form.** Save a **screenshot of the QR code** on the mobile device so you can easily find the form.

**A guide to help you take great photos!**

| Steps | Information |
| --- | --- |
| ***Step 1: Stay Safe***  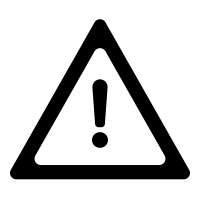 | Remember to **stay safe.**   - Always wear a **helmet** when cycling. - Ask **a family member or support person** to help you take photos if you are riding (see ***Step 3***). - **Plan ahead**: Do you need to pull over, slow down or stop to take your photo safely? - **Watch out** for other cyclists and people walking. - Stay away from busy **traffic**. |
| ***Step 2: Ask Permission***  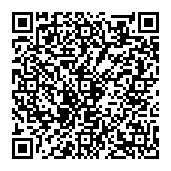  [**https://redcap.link/---**](https://redcap.link/---) | Remember to **be** **responsible**   - Asking people who want to be in your photo to fill out a **photo consent form**. - Fill out the form **first**, *then* take the photograph. - Check if they want their **face** (which can be easily recognised) in the photo. |
| ***Step 3: Be A Director***  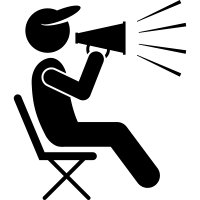 | We want *you* to **direct** how the photos are taken.  This means;   - Taking the photo **yourself** when it’s safe to do so. - Asking a family member or a support person to take the photo when you’re cycling or in a busy area. - Tell them how you would like the photo to be taken.   *for *director* tips and tricks see **Page 4**. |
| ***Step 4: Take photos!***  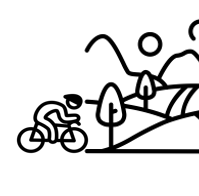 | Take as many photos as you like before your interview.  You can use old photos that you, family or friends took in the past.  We think it’s useful to have at least 10 photos to choose from. |
| ***Step 5: Decision Time***  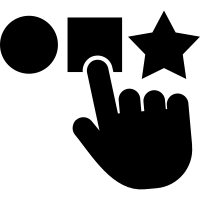 | Choose **three photos** to send to the researchers.  Tell us about each photo in one or two sentences. We’d like to hear about:   - Why you took this photo - What’s happening in this photo   If someone helped you take the photo, tell us who helped and what you directed them to do!  If it’s an older photo, tell us when it was taken and why you have included it. |
| ***Step 6: Send email***  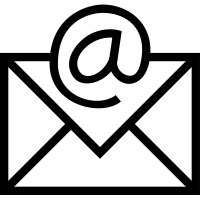 | Ask a family member or support person to help you send the **email** to:  [cyclink-codesign@unimelb.edu.au](mailto:cyclink-codesign@unimelb.edu.au)  Don’t forget to include;   - Your final three photos - A short sentence about the photo(s) - The names of other people in the photo(s) |

**Need more help?**

If you would like further information about the photo-interview, please contact the researchers; John Carey (Phone: NUMBER or email: [jjcarey@student.unimelb.edu.au](mailto:jjcarey@student.unimelb.edu.au)) or Dr Rachel Toovey (Phone: NUMBER or email: [r.toovey@unimelb.edu.au](mailto:r.toovey@unimelb.edu.au)).

**Director’s top tips for taking photos**

| **Lighting**  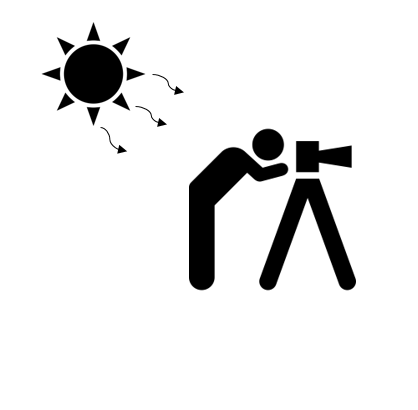 | Photos are best taken when there’s enough light.  Switch on the lights if you’re indoors.  Make sure the light (e.g. sun or window) is **behind** the person taking the photograph. |
| --- | --- |
| **Photo layout**  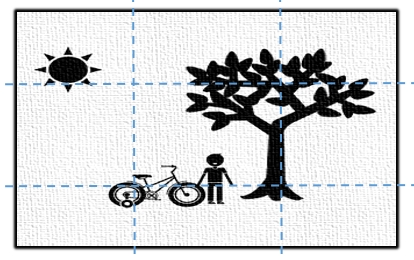 | What is your photo about?  It might be you, your bike, or a place.  Try keep the most important part of the photo in the middle.  Try out different views and angles;   - from in front - from the side - below - overhead - at an angle - close up and from a distance   Can you frame the photo with a tree or building? |
| **Decide**  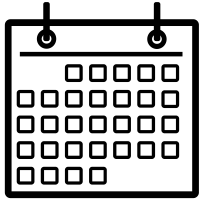 | Plan when you’ll take the photographs.  Pick a time that works for you and your family.  The morning time and late afternoon (‘golden hour’) are good times for outdoor light. |
| **Ask Permission**  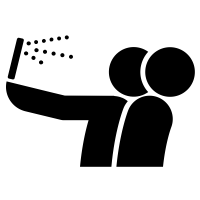 | Make sure everyone in the photo has given their permission to be photographed.  Ask everyone in the photo to fill out and sign the consent form. |

# File folder information sheet


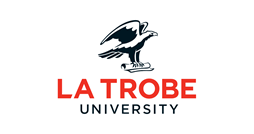

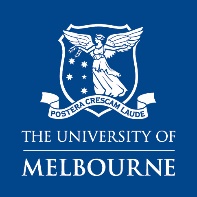


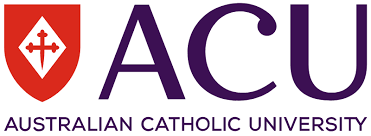


**Young person file folder for more help**

Some young people and families find it helpful to keep a record of the photos they take or have a little more direction on potential ideas.

This folder with different plastic files can be helpful for keeping track.

| **Keep a folder**  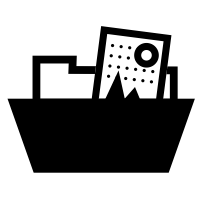 | It can be useful to keep a folder to keep track of your photos.  We suggest having 4 plastic pockets with photos that capture ideas like:   1. Cycling makes me **feel**… 2. **Places** that I go cycling… 3. What makes cycling **easy**… 4. What makes cycling **a challenge**… |
| --- | --- |

# Worked Example


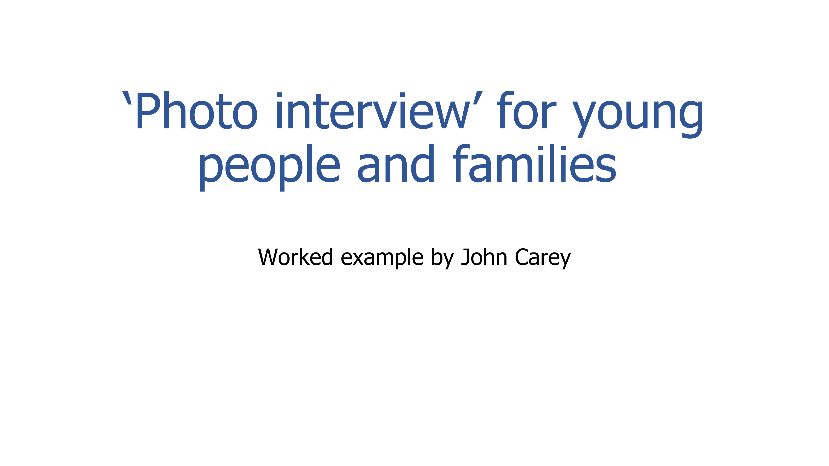


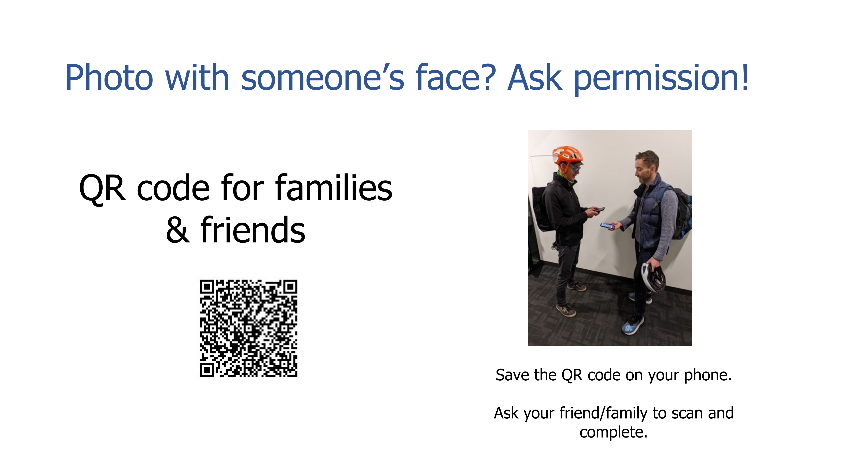


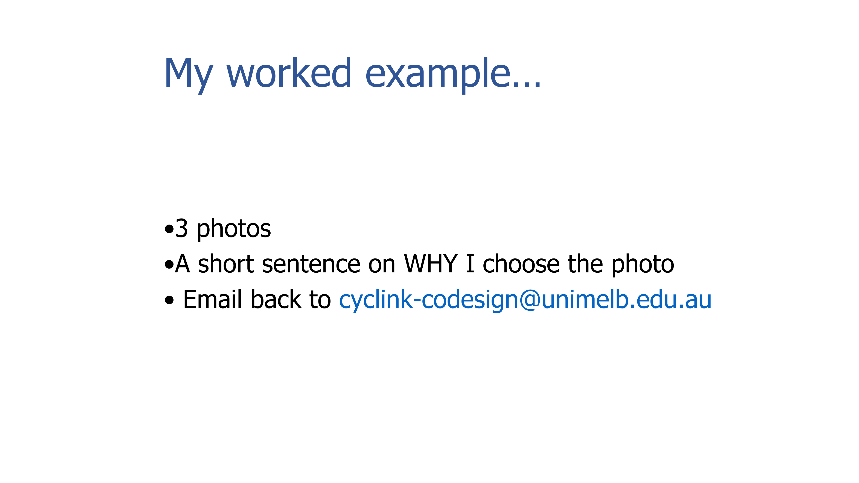


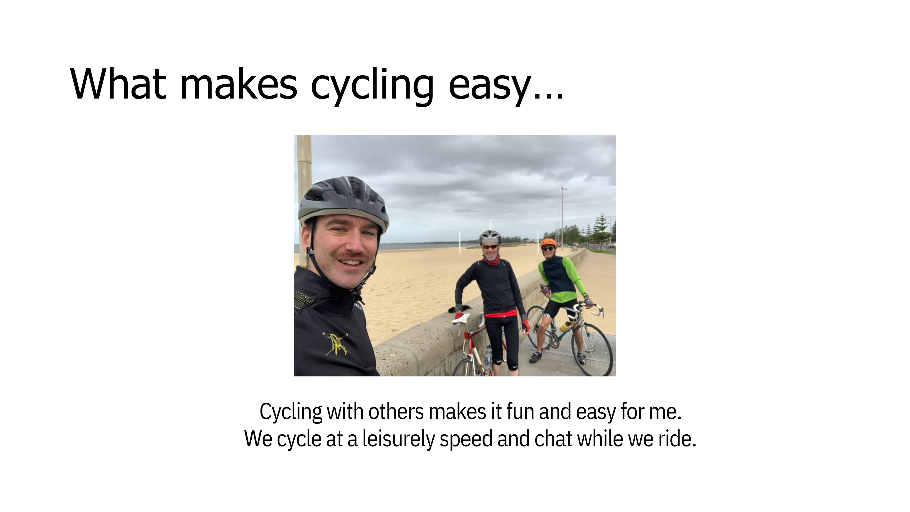


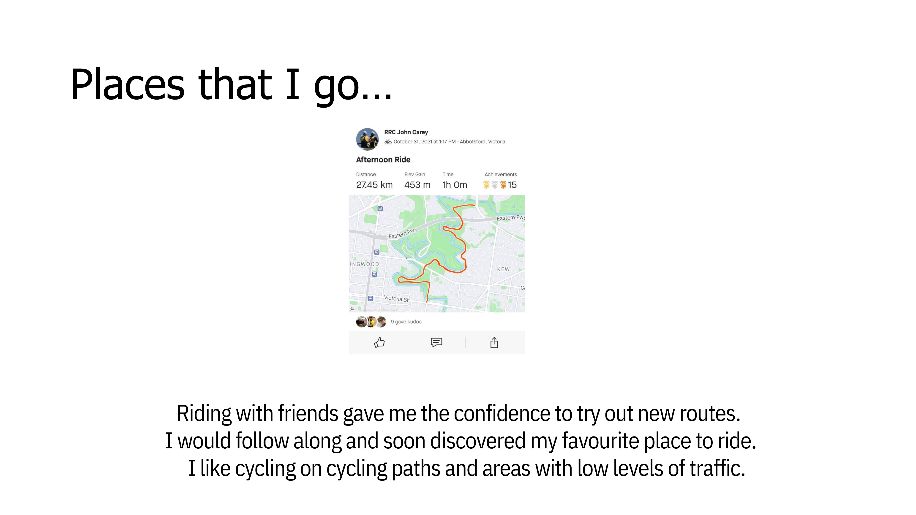


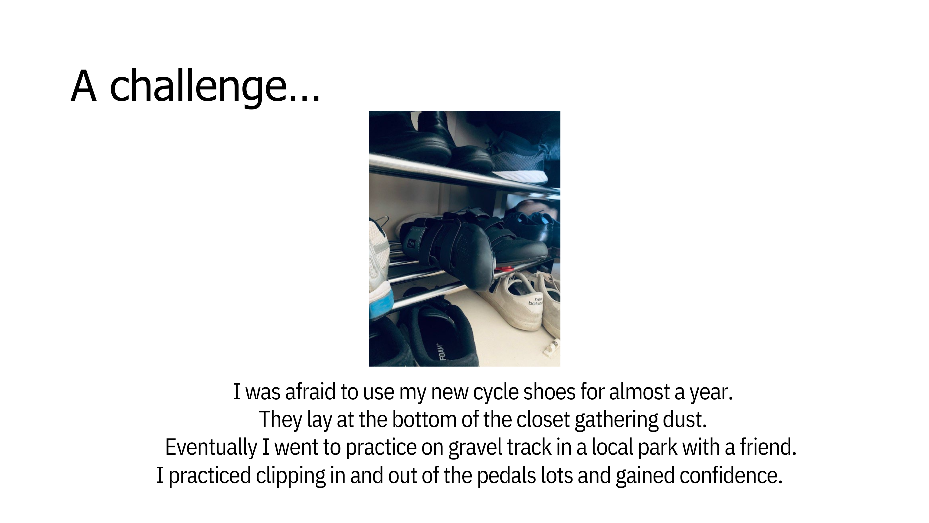


# Communication support partner briefing

**Email brief to go out to parent/caregiver prior to interview**

Thank you for agreeing to support [CHILD/YOUNG PERSON’S NAME] during the photo-interview. We want to hear about cycling from the young person’s perspective in these interviews.

On the day of the interview, you may need to help [YOUR CHILD] access the Zoom link. We would also like you to sit beside [YOUR CHILD/YOUNG PERSON’S NAME] at the start of the interview so we can work out if you need to be present throughout our chat.

*For children and young people who indicate that they have complex communication needs, intellectual disability or require adult support…*

As a support person, your role is to help [YOUR CHILD/ YOUNG PERSON’S NAME] understand our questions and clarify [YOUR CHILD/ YOUNG PERSON’S NAME]’s response.

For example, you might assist [YOUR CHILD/ YOUNG PERSON’S NAME] to use their communication device or rephrase our questions with familiar language.

We might ask you to help repeat their message if their speech is unclear or difficult to understand. In such cases:

- If you think you know what [YOUR CHILD] is saying you might say “I think [he/she/they] is saying that “[he/she/they] likes....” rather than expressing your perspective of “[he/she/they] likes......”.
- If you don’t know what [YOUR CHILD] is saying, you should make this clear.

We will always direct the questions towards [YOUR CHILD] and wait to see if [he/she/they] responds first. If [he/she/they] does not reply, and we are satisfied that we have allowed adequate time, we will let you know that we would be interested in hearing your interpretation by saying your name. After you speak, we might check back in with [CHILD/YOUNG PERSON’S NAME] to get their opinion.

Throughout the interview, you are welcome to add a clarifying statement or your own interpretation of their key message if you feel it’s appropriate.

#
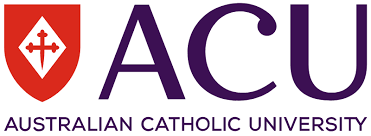
 Advisory group preparation material (meeting 1)


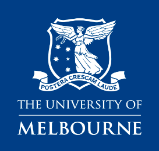

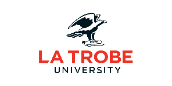


**Welcome to the young person’s group!**

Our first group is about helping a girl called Edith to get started with cycling. Her goal is to meet others, get fit and have fun.

We’ll start the group by saying hello.

Then, we’ll write Edith’s story together.

**How to get ready:**

**1. Think about your favourite place to ride.**


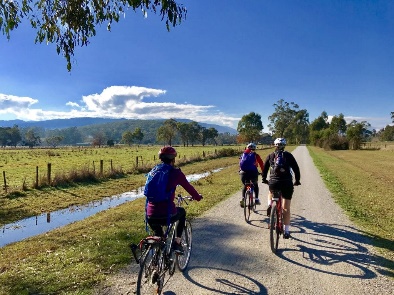


**2. Complete your cycling comic strip.**

| *Once upon a time in…*  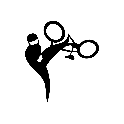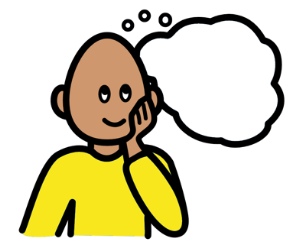 | **Then I…** | *And I rode towards my goal of…*  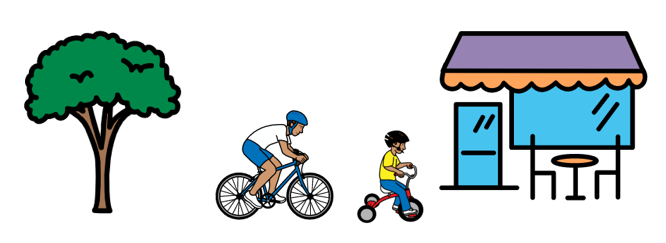 |
| --- | --- | --- |

You can do this by writing down memories about your cycling story.

**See you on Monday 28^th^ November at 5-6pm!**

Zoom Link: ___

*Picture Communication Symbols^®^ (PCS) is a trademark of Tobii Dynavox LLC.*

*All rights reserved. Used with permission.*

# Content for sharing on digital display tool


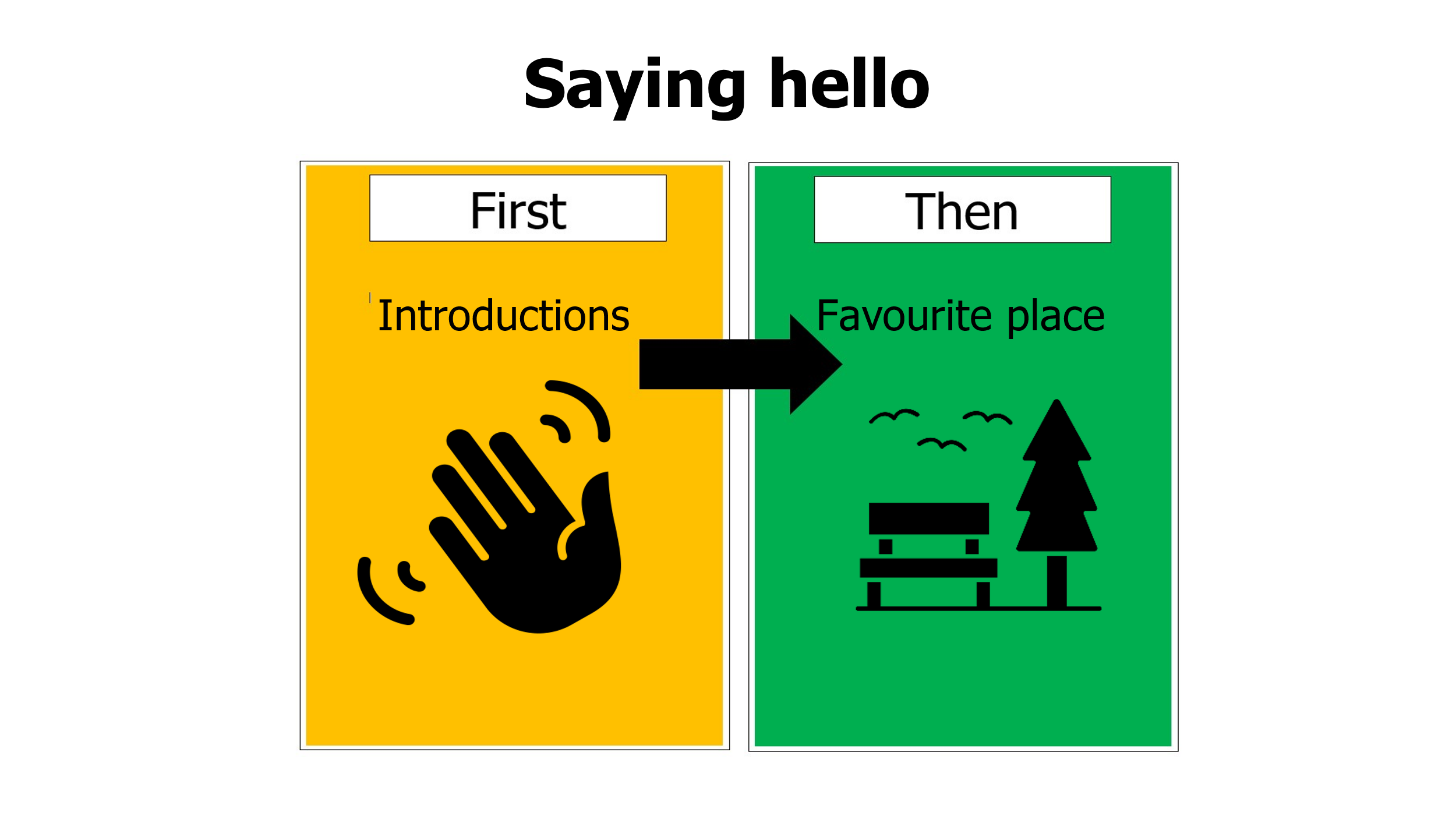


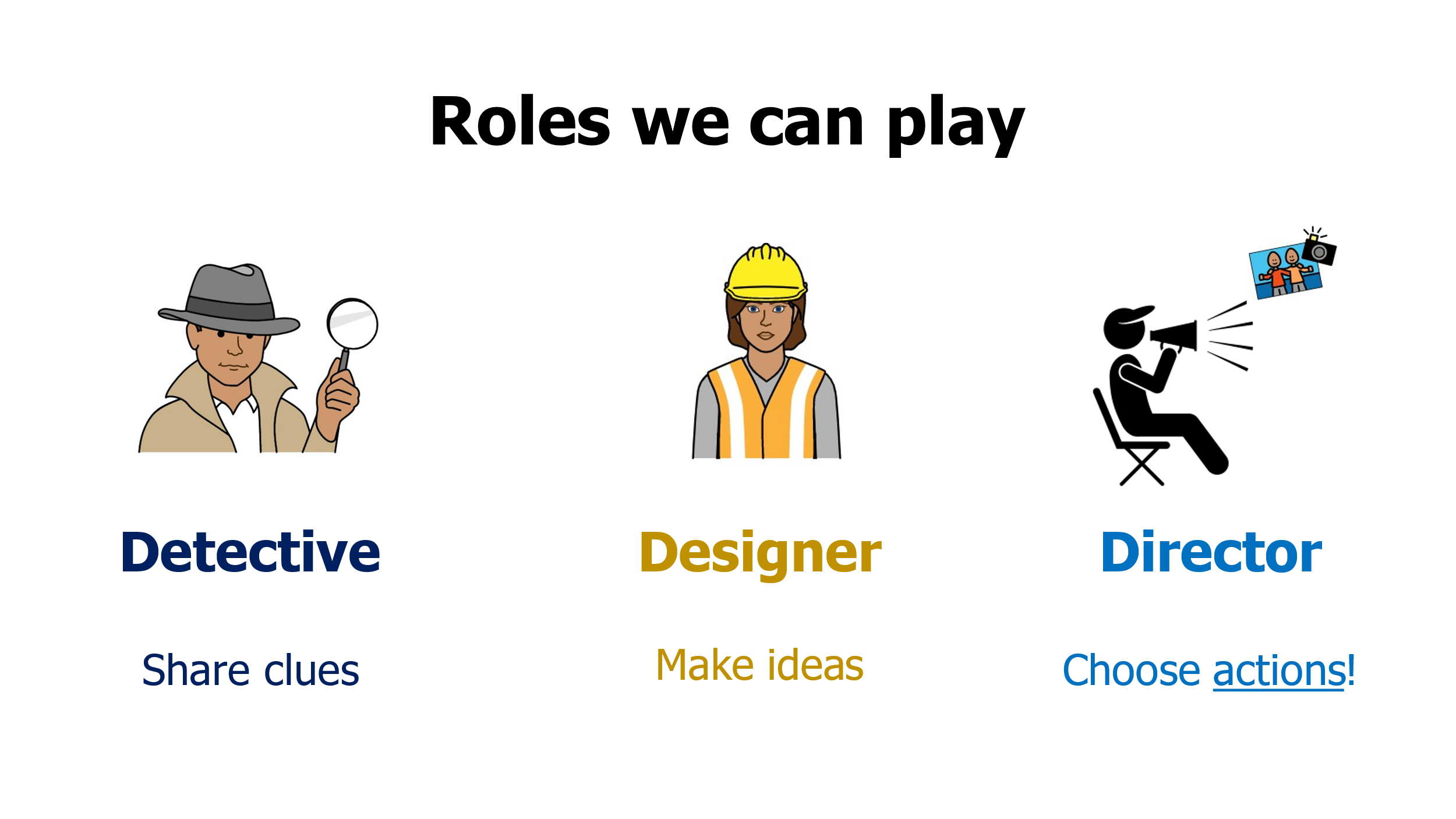


*Picture Communication Symbols^®^ (PCS) is a trademark of Tobii Dynavox LLC.*

*All rights reserved. Used with permission.*


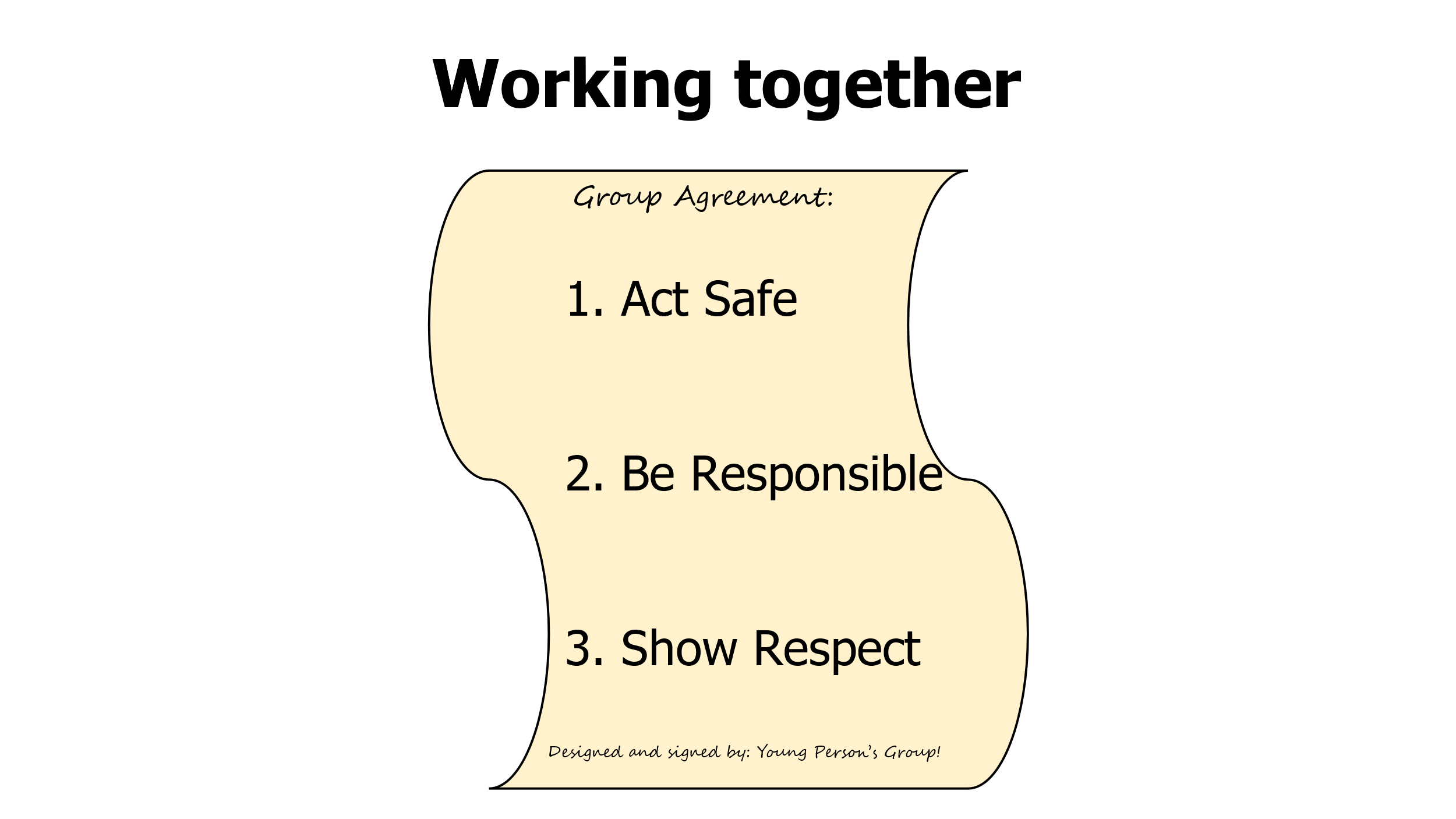


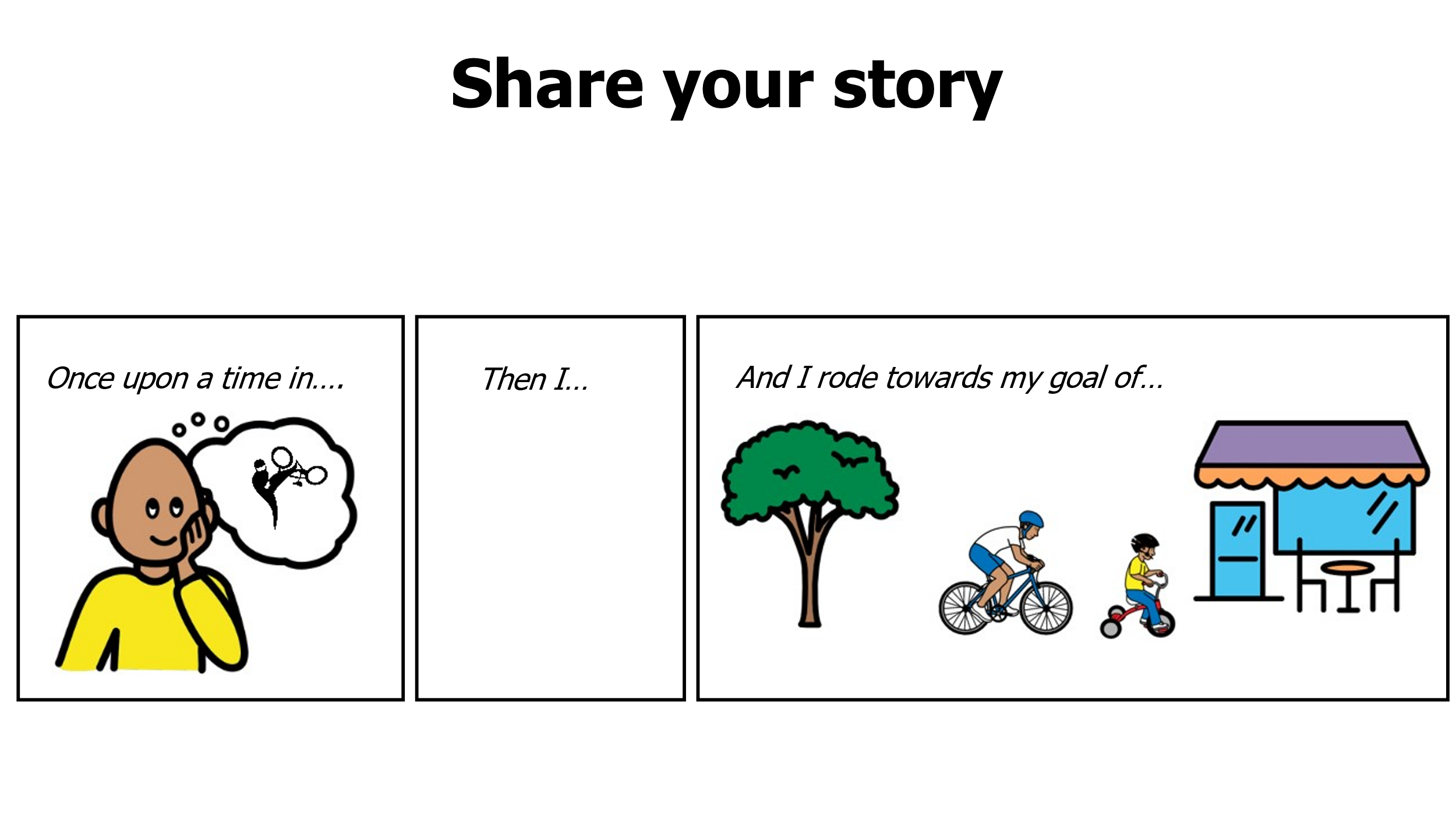


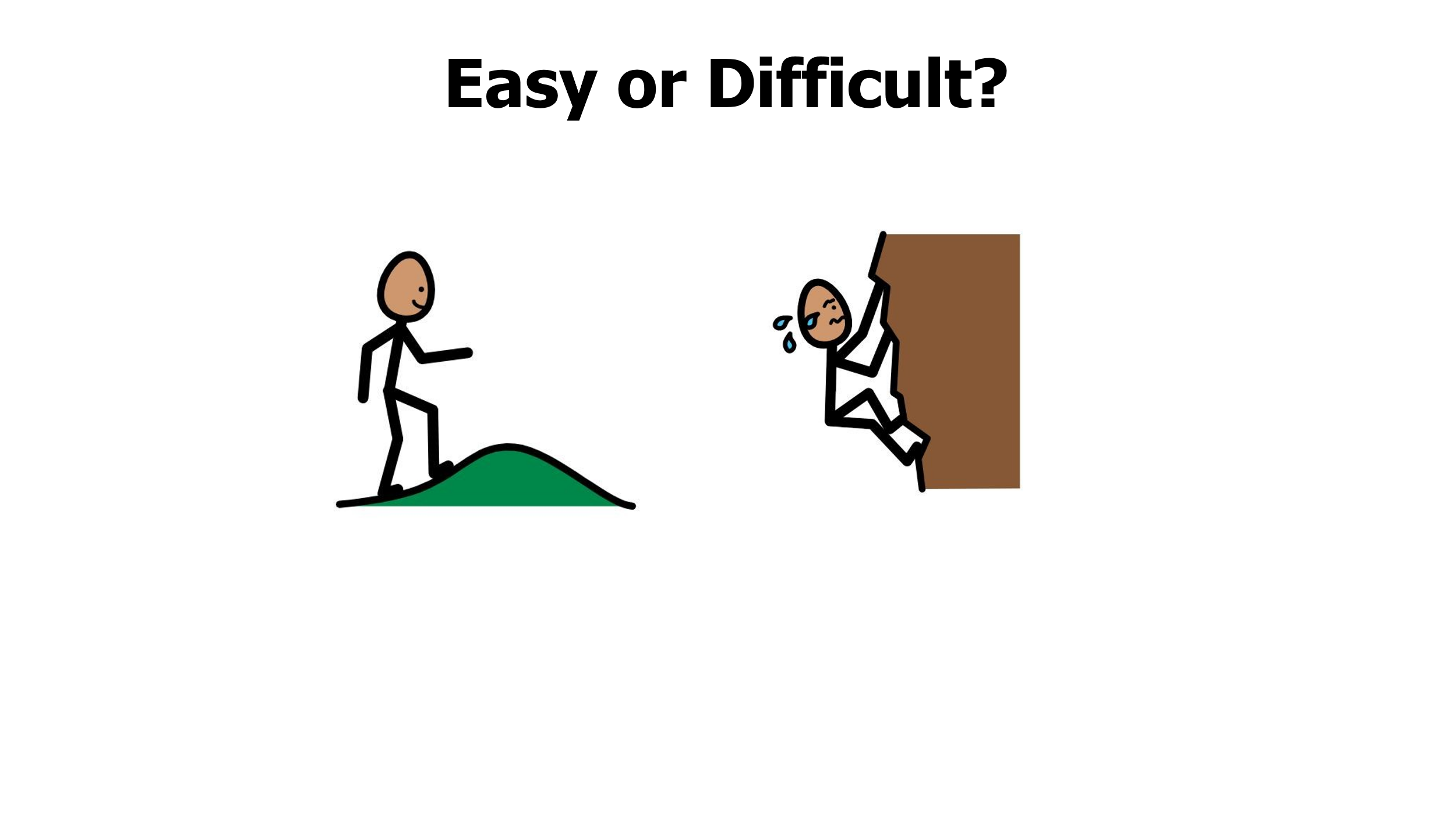


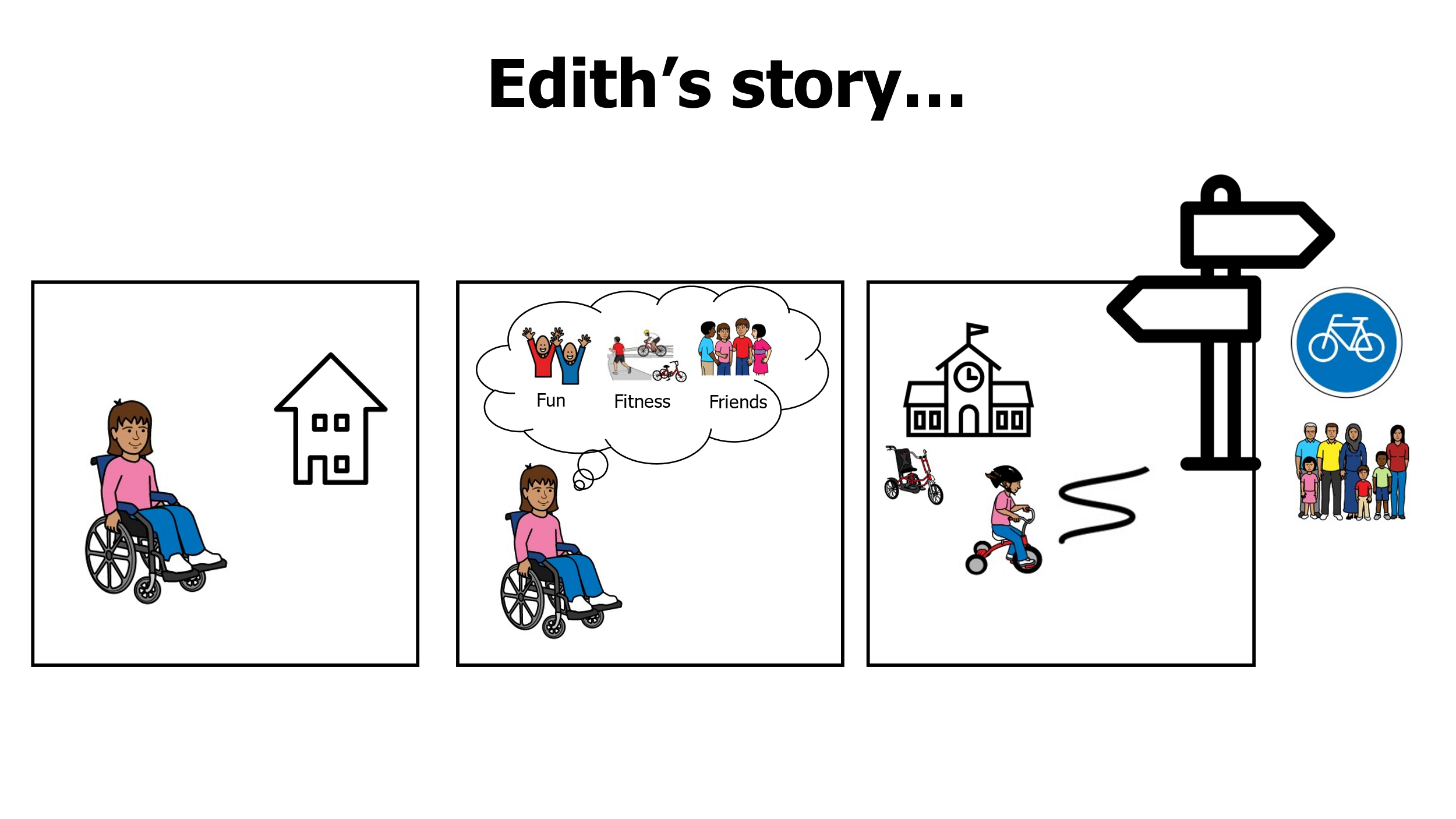


*Picture Communication Symbols^®^ (PCS) is a trademark of Tobii Dynavox LLC.*

*All rights reserved. Used with permission.*
